# Precision Drug Delivery of LY-11h for Acute Myeloid Leukemia Treatment Using Machine Learning-Assisted Hot Melt Extrusion and 3D-Printed Technologies

**DOI:** 10.3390/pharmaceutics18081002

**Published:** 2026-08-13

**Authors:** Lianghao Huang, Danhui Li, Tiantian Yang, Weiwei Yang, Minqing Zhu, Xia Zhao, Jiaxiang Zhang

**Affiliations:** 1 Key Laboratory of Marine Drugs, Ministry of Education, School of Medicine and Pharmacy, Ocean University of China, Qingdao 266003, China; 2 Qingdao Institute of Bioenergy and Bioprocess Technology, Chinese Academy of Sciences, Qingdao 266101, China; 3 Shandong Energy Institute, Qingdao 266101, China; 4 Qingdao New Energy Shandong Laboratory, Qingdao 266101, China; 5 Material Characterization, Thermo Fisher Scientific, Shanghai 201203, China; 6 Pharmaceutical Products Research and Development Center, Marine Biomedical Research Institute of Qingdao, Qingdao 266137, China

**Keywords:** amorphous solid dispersion, hot-melt extrusion, additive manufacturing, process analytical technology, machine learning

## Abstract

**Background**: Acute myeloid leukemia (AML) is a heterogeneous and aggressive hematologic malignancy, and LY-11h is a novel acylhydrazide-based histone deacetylase inhibitor with promising therapeutic potential for AML. However, its poor aqueous solubility, limited intestinal dissolution, and narrow therapeutic window hinder oral formulation development and motivate the development of dosage forms with flexible dose-design capabilities. Herein, an integrated hot-melt extrusion (HME)–fused deposition modeling (FDM) strategy was developed to convert LY-11h into printable amorphous solid dispersion (ASD) dosage forms. **Methods**: HPMC-AS was used as a pH-responsive carrier to enhance intestinal release while restricting premature gastric release, and HPC-EF was incorporated to improve filament processability. Single-factor and DoE studies identified critical formulation and process variables and established formulation–process–property relationships, while machine learning further modeled nonlinear interactions and guided optimization. In-line near-infrared spectroscopy combined with polarized light microscopy enabled real-time monitoring of LY-11h amorphization and melt homogenization during HME. **Results**: ExtraTrees and Bagging models showed promising predictive performance for key filament properties, and PAT-stage validation confirmed strong agreement with experimental values. The 15 DoE-designed ASD filaments were successfully fabricated into FDM-printed tablets with reproducible geometry. Equilibrium-solubility and in vitro dissolution studies demonstrated enhanced intestinal-pH solubility and reproducible pH-responsive release. **Conclusions**: Collectively, these findings establish a technological proof of concept for the manufacture of LY-11h dosage forms with adjustable formulation and geometric attributes. Further in vivo pharmacokinetic studies are required to determine whether these manufacturing capabilities translate into predictable dose–exposure relationships and individualized dose control.

## 1. Introduction

Acute myeloid leukemia (AML) is the most prevalent form of acute leukemia in adults and arises from the malignant transformation of myeloid progenitor cells [1]. With advances in understanding the molecular pathogenesis of AML, targeted agents against FLT3, IDH1/2, and BCL-2 have been approved [2]. However, therapeutic resistance and disease heterogeneity remain major clinical challenges, supporting the continued development of alternative therapeutic strategies, including histone deacetylase (HDAC) inhibition. Moreover, interpatient variability in disease characteristics and treatment tolerance creates a need for flexible therapeutic strategies and dosage forms that can accommodate individualized dose adjustment [3,4,5,6].

Compound LY-11h is a novel acylhydrazide-based histone deacetylase inhibitor (HDACi) developed for the treatment of AML and was designed to overcome limitations associated with conventional HDAC inhibitors, particularly nonspecific responses related to Michael acceptor moieties [7]. LY-11h has been reported to effectively inhibit HDAC enzymatic activity, resulting in histone hyperacetylation and subsequently inducing cell-cycle arrest, cell differentiation, and apoptosis [7]. However, two formulation-related challenges complicate its development as an oral dosage form. First, its hydrophobicity results in limited solubility in intestinal fluid environments, which may restrict oral absorption and contribute to variability in drug exposure. Second, its narrow therapeutic window leaves only a limited margin between therapeutically effective and potentially toxic doses, increasing the importance of accurate and flexible dose adjustment. Conventional fixed-dose oral formulations may therefore be insufficiently adaptable to the differing dose requirements encountered during individualized treatment. Accordingly, there is a strong need to develop advanced drug-delivery systems that can improve the apparent solubility of LY-11h while enabling flexible dose modulation and controlled release and thereby provide a technological basis for the subsequent development of individualized LY-11h therapy.

Amorphous solid dispersion (ASD) is a widely used formulation strategy for enhancing the dissolution performance of poorly water-soluble drugs [8,9]. By dispersing the drug in an amorphous state within a polymeric carrier, ASD systems can increase apparent solubility, improve dissolution rate, and reduce drug crystallization during storage and release [10]. Hydroxypropyl methylcellulose acetate succinate (HPMC-AS) is an enteric polymer that remains largely insoluble under acidic gastric conditions and dissolves at grade-dependent intestinal pH values, thereby limiting premature gastric release and promoting drug release after gastric emptying [11,12]. The L-, M-, and H-substitution types exhibit nominal dissolution thresholds of approximately pH ≥ 5.5, ≥6.0, and ≥6.5–6.8, respectively, whereas the F and G suffixes denote fine-powder and granular physical grades [13,14]. In the present study, LY-11h release was intended to occur primarily in the mid-to-distal small-intestinal environment, represented experimentally by simulated intestinal fluid at pH 6.8 [15]. Although the H-type polymer was nominally aligned with this pH range, LG, MG, HG, and HF were initially screened because polymer selection for an HME–FDM formulation also depends on drug–polymer interactions, amorphization, extrusion behavior, ASD stability, and filament performance [16].

Additive manufacturing, commonly known as 3D printing, has become increasingly accessible for flexible pharmaceutical manufacturing, and fused deposition modeling (FDM) has emerged as a promising technique for pharmaceutical dosage-form fabrication [17,18,19]. Unlike conventional manufacturing approaches that generally produce dosage forms in a limited number of fixed strengths, FDM enables dosage-form attributes to be modified through digital design and controlled material deposition. By adjusting dosage-form geometry, infill density, shell structure, and drug-loaded filament composition, FDM enables flexible control of nominal dose and in vitro release behavior [20,21,22,23,24,25]. These manufacturing capabilities may therefore support the future development of variable-dose LY-11h products for individualized therapeutic use. Nevertheless, FDM printing relies heavily on the quality of drug-loaded filaments, which should possess suitable uniformity, mechanical strength, flexibility, thermal stability, and melt behavior to ensure continuous feeding and stable printing. The development of a printable LY-11h ASD filament therefore requires simultaneous consideration of drug amorphization, polymer composition, mechanical integrity, and thermal-process suitability.

Design of experiments (DoE), process analytical technology (PAT), and machine learning (ML) provide complementary tools for formulation and process development [26,27,28]. DoE enables systematic evaluation of the main and interactive effects of formulation and process variables, whereas PAT permits real-time monitoring of material transformation during manufacturing [28,29]. Near-infrared spectroscopy can provide rapid and nondestructive information on composition and solid-state changes during hot-melt extrusion [30], while ML can capture nonlinear formulation–process–property relationships [31]. DoE is particularly useful for interpreting factor effects within a predefined experimental space, whereas ML can complement this analysis by identifying more complex relationships across combined formulation and process datasets. Their integration with PAT may therefore provide a more comprehensive understanding of material attributes, processing conditions, and filament performance while reducing reliance on trial-and-error development.

Accordingly, this study aimed to develop an integrated HME–FDM platform assisted by DoE, PAT, and ML for the preparation of LY-11h ASD dosage forms. HPMC-AS was investigated as a pH-responsive carrier, while HPC-EF was incorporated to improve filament processability. Formulation and process variables were initially screened, followed by Box–Behnken evaluation of extrusion temperature, screw speed, and drug loading. ML models were developed to predict filament maximum force and distance at break, and an in-line NIR-based PAT system was established to monitor LY-11h amorphization and melt homogenization during HME. The resulting ASD filaments were subsequently evaluated for FDM printability, solid-state characteristics, equilibrium solubility, and pH-responsive drug release. Collectively, this work establishes a technological proof-of-concept platform for improving the intestinal delivery of LY-11h and enabling the flexible manufacture of dosage forms with adjustable formulation and geometric attributes, thereby supporting subsequent pharmacokinetic evaluation and the future development of individualized LY-11h treatment strategies.

## 2. Materials and Methods

### 2.1. Materials

**Figure 1:**
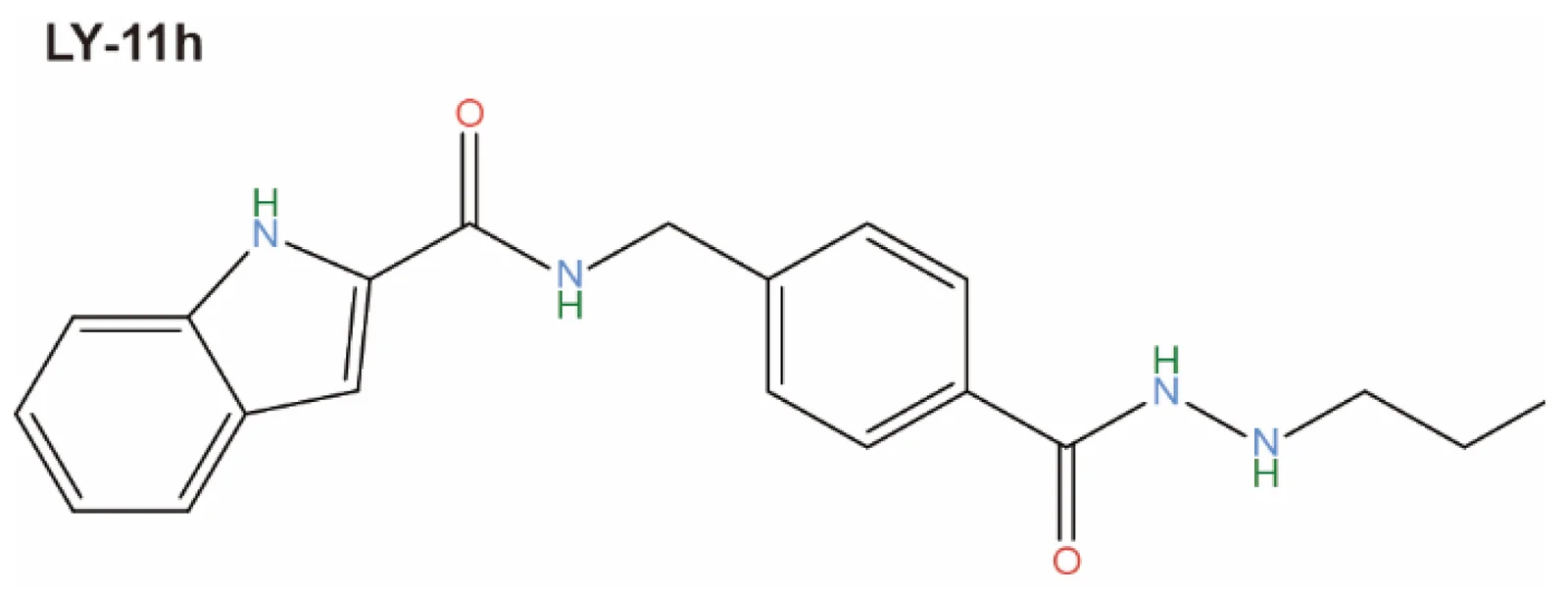
The structure formula of LY-11h.

LY-11h, a histone deacetylase inhibitor with the molecular formula C_20_H_22_N_4_O_2_, was synthesized by the research group at Ocean University of China. Its chemical structure is shown in Figure 1 [7]. Hydroxypropyl methylcellulose acetate succinate (HPMC-AS) HF was purchased from Taian Ruitai Cellulose Co., Ltd. (Taian, China). HPMC-AS LG, MG, and HG were kindly supplied by Japan Vam & Poval Co., Ltd. (Osaka, Japan). Hydroxypropyl cellulose EF (HPC-EF) was purchased from Ashland (Shanghai, China). All other chemicals, solvents, and reagents used in this study were of analytical or HPLC grade.

### 2.2. Methods

#### 2.2.1. Preparation of LY-11h-Loaded Filaments via HME Process

LY-11h-loaded filaments were prepared using an 11-mm co-rotating twin-screw extruder (Process 11 HYG, Thermo Fisher Scientific, Shanghai, China). An automated feeder (Thermo Fisher Scientific, Shanghai, China) was used to deliver the PM to the feeding zone (zone 1), with the feeding rate adjusted to about 3 g/min. The screw configuration and temperature profile were adjusted according to the experimental design. During extrusion, process parameters including barrel temperature, screw speed, and torque were recorded. The extrudates were collected on a conveyor belt to maintain filament straightness. Filament diameter was monitored during collection using a micrometer. Selected filaments were milled and passed through a 40-mesh sieve for solid-state and dissolution characterization. The milled extrudates were defined as EXT samples. All samples were stored in a desiccator before further analysis.

#### 2.2.2. Preliminary Formulation and Process Development Using Single-Factor Experiment Methodologies

Single-factor experiments were conducted as a preliminary screening step to define a practical formulation and processing window for producing LY-11h-loaded filaments suitable for downstream FDM printing. The overall objectives were to identify unsuitable formulation or processing conditions, determine variables that could be fixed in subsequent studies, and select the factors and ranges requiring further multivariate optimization.

The screening conditions were evaluated using an integrated set of optimization criteria, including: (i) the amorphous state of LY-11h, as assessed by DSC, PXRD, and PLM; (ii) continuous extrusion and the formation of filaments with uniform morphology; (iii) adequate maximum force and distance at break for filament handling, feeding, and printing; and (iv) maintenance of the intended pH-dependent solubility behavior, characterized by limited apparent solubility at pH 1.2 and enhanced LY-11h solubility at pH 6.8.

The investigated variables included drug loading, HPC-EF content, HPMC-AS grade, extrusion temperature, number of mixing sections, and screw speed. Drug loading was set at 5%, 10%, 30%, and 50% *w*/*w*. For the drug-loading investigation, the extrusion temperature and screw speed were fixed at 140 °C and 100 rpm, respectively, while HPMC-AS HF, 5% (*w*/*w*) HPC-EF, and a screw configuration containing two mixing sections were used. HPC-EF content was set at 2.5%, 5%, 10%, 30%, and 50% *w*/*w*. Four HPMC-AS grades were compared, including HF, LG, MG, and HG. Extrusion temperature was set at 120, 140, 160, and 180 °C. The number of mixing sections was set at 0, 1, and 2. Screw speed was set at 50, 100, and 200 rpm.

Based on the integrated screening results, HPMC-AS HF, 5% HPC-EF, and two mixing sections were selected as fixed conditions because they provided the most favorable overall balance of amorphous-state maintenance, filament mechanical performance, morphology, and processability. Drug loading, extrusion temperature, and screw speed were retained as the independent variables for subsequent DoE optimization because they produced pronounced changes in filament solid-state properties, mechanical performance, and extrusion behavior and were therefore expected to exhibit potentially important interactive effects.

#### 2.2.3. Comprehensive Process Development Using DoE Methodologies

A three-factor, three-level Box–Behnken design was employed to evaluate the individual and interactive effects of extrusion temperature, screw speed, and drug loading on the critical quality attributes of LY-11h-loaded filaments [32,33]. A total of 15 experimental runs, including three independently prepared center-point replicates at 140 °C, 150 rpm, and 17.5% (*w*/*w*) drug loading, were performed. Each response was measured in triplicate (n = 3). For each experimental run, the mean value obtained from the three replicate measurements was used as the response value for DoE modeling. For graphical clarity, representative curves are shown for selected characterization results. Process reproducibility was evaluated using the three independently prepared center-point runs by calculating the mean, standard deviation, and coefficient of variation for each response. Model fitting and response-surface analysis were performed using Design-Expert 8.0.6 software. Different response transformations were compared, and the statistically significant transformation providing the highest R^2^ was selected for response-surface analysis. Overall model significance was evaluated by analysis of variance (ANOVA), with *p* < 0.05 considered statistically significant.

Distance at break and maximum force were selected as complementary indicators of filament mechanical performance. Distance at break reflected filament flexibility and resistance to brittle fracture, whereas maximum force represented the mechanical robustness required for filament handling, feeding, and FDM printing. These two responses were evaluated jointly because successful printing requires an appropriate balance between filament flexibility and strength.

Amorphization (%) was defined as the NIR-predicted amorphous fraction of LY-11h. The physical mixture and a fully amorphous extrudate were assigned as the 0% and 100% amorphization references, respectively. The absence of residual crystalline LY-11h in the fully amorphous reference was confirmed by DSC, PXRD, and PLM. Calibration samples were prepared by mixing the physical mixture and fully amorphous extrudate at predefined proportions. The NIR spectra were subjected to second-derivative preprocessing, and the spectral response at 4771.0 cm^−1^ was used to establish a calibration model using TQ Analyst 9 software (Thermo Fisher Scientific). The same calibration model was applied to formulations containing 5.0%, 17.5%, and 30.0% LY-11h after confirming that the characteristic spectral response was consistently observed across the investigated drug loadings. Each DoE sample was measured in triplicate, and the mean NIR-predicted amorphous fraction was used as the response value. DSC, PXRD, and PLM were used as complementary methods to assess residual crystallinity.

The glass-transition temperature (Tg) was determined from the step transition in the DSC thermogram using the instrument software. Tg was included as an indicator of molecular mobility and the potential physical stability of the amorphous drug–polymer matrix. Comparatively higher Tg values within the investigated design space were considered favorable because they generally indicate reduced molecular mobility. However, no absolute Tg acceptance threshold was assigned, and Tg was not used alone as evidence of long-term storage stability.

Extrusion torque was recorded as the average value during the steady-state extrusion stage. It was selected as an in-process indicator of melt-flow resistance, formulation processability, mixing behavior, and mechanical load on the extruder. Excessively high torque was considered undesirable because it may indicate inadequate melt flow and increased equipment load, whereas very low torque may reflect insufficient melt resistance or mixing. Therefore, torque was evaluated within the experimentally feasible operating range, with the objective of maintaining stable extrusion rather than minimizing torque without constraint.

No fixed numerical acceptance limits or independent desirability targets were assigned to the response variables. Instead, the response-surface models were used to evaluate the effects and interaction trends of the investigated factors and to identify a practical formulation and processing region that provided balanced filament strength and flexibility, a high NIR-predicted amorphous fraction, a comparatively favorable Tg, and stable extrusion torque.

#### 2.2.4. Investigation of Filament Mechanical Properties Using ML Methods

The machine-learning workflow was designed to systematically evaluate regression algorithms with different learning mechanisms [34]. All machine-learning analyses were performed in Python (version 3.10) using the scikit-learn library. The ExtraTreesRegressor and BaggingRegressor implementations from the sklearn.ensemble module were used to predict maximum force and distance at break, respectively. Data preprocessing, feature standardization, model fitting, and leave-one-out cross-validation were implemented within the scikit-learn modeling workflow. The complete dataset consisted of 102 individual mechanical-test observations, including 57 observations from the single-factor screening studies and 45 observations from the 15-run Box–Behnken design. Three replicate measurements obtained under each experimental entry were retained as separate observations to preserve the experimentally observed variability in filament mechanical performance.

The ML analysis was introduced as a complementary approach rather than as a replacement for the DoE model. The Box–Behnken design evaluated extrusion temperature, screw speed, and drug loading within a predefined experimental space using quadratic response-surface models. In contrast, the ML dataset integrated observations from both the single-factor screening and DoE studies and included additional formulation and process variables, namely HPC-EF content and the number of mixing sections. ML algorithms were therefore used to explore nonlinear and non-monotonic formulation–process–property relationships without imposing a predefined polynomial structure and to provide predictive and feature-importance analyses for filament mechanical performance.

The investigated algorithms covered four representative model families: linear regression models, kernel-based and nearest-neighbor models, single decision-tree models, and ensemble tree-based models. Linear models were included as baseline algorithms to assess whether the relationships could be described by simple linear trends. Kernel-based, nearest-neighbor, decision-tree, and ensemble algorithms were evaluated because they can describe more complex nonlinear relationships among drug loading, HPC-EF content, extrusion temperature, screw speed, and the number of mixing sections. The input variables consisted of drug loading, HPC-EF content, extrusion temperature, screw speed, and number of mixing sections, while maximum force and distance at break were used as prediction targets. Before model training, categorical variables were numerically encoded, and feature standardization was applied within the modeling pipeline for algorithms sensitive to variable scaling.

Leave-one-out cross-validation was used because of the limited dataset size. In each cross-validation iteration, 101 observations were used for model training, and the remaining observation was used for validation, until every observation had served once as the validation sample. Model performance was compared using the coefficient of determination (R^2^), root mean square error (RMSE), mean absolute error (MAE), and mean absolute percentage error (MAPE). The final models were selected based on their overall cross-validation performance and prediction stability rather than on a single metric [35,36].

ExtraTrees regression was selected for maximum-force prediction, whereas Bagging regression was selected for distance-at-break prediction. Feature-importance analysis was subsequently used to compare the relative contributions of formulation and process variables and to examine whether the data-driven trends were consistent with those identified by the DoE response-surface analysis.

#### 2.2.5. Continuous Manufacturing Process Setup and PAT Implementation

An in-line near-infrared spectroscopy system was integrated with the twin-screw extruder to monitor material transformation during HME [37]. The NIR probe was mounted on the extruder barrel (Die) and positioned in direct contact with the molten material through a sapphire window. Spectra were collected every 30 s over the range of 4000–10,000 cm^−1^, with 32 scans averaged for each spectrum and a spectral resolution of 8 cm^−1^. During extrusion, screw speed, barrel temperature, feeding conditions, and torque were recorded. Extrudate samples were collected at selected process stages for off-line characterization. The NIR spectral changes were compared with polarized light microscopy observations to evaluate drug amorphization and melt homogenization during HME.

#### 2.2.6. 3D Designs and Printing

Cylindrical tablet models were designed using 3D Builder (version 20.0.4.0) and sliced using CURA (version 5.13) software. The tablets were designed with five outer shells, 50% infill, and open top and bottom faces. Printing was performed using an Ender-3 S1 Pro FDM printer equipped with a 0.4 mm nozzle. The nozzle temperature, build-plate temperature, and printing speed were set at 210 °C, 50 °C, and 50 mm/s, respectively. After printing, the tablets were evaluated for printing success rate, diameter, thickness, mass, and surface morphology. Dimensional measurements were performed using a digital caliper, and tablet morphology was observed using optical microscopy.

To quantify printing success, a total of 20 printing attempts were performed using the optimized filament. A printing attempt was considered successful when the filament was continuously fed and extruded throughout the entire printing process without fracture or interruption, resulting in a complete tablet with the intended overall geometry. An attempt was classified as unsuccessful when filament breakage interrupted material feeding or resulted in an incomplete tablet. The printing success rate was calculated as the number of successfully completed tablets divided by the total number of printing attempts and multiplied by 100.

After printing, the tablets were evaluated for printing success rate, diameter, thickness, mass, and surface morphology. Dimensional measurements were performed using a digital caliper, and tablet morphology was observed using optical microscopy.

#### 2.2.7. Characterization and Evaluation of Printed LY-11h Dosages

The solid-state and functional characterization methods are described in the Supplementary Information, including thermalgravimetric analysis (TGA) (NETZSCH Gerätebau GmbH, Bavaria, Germany), differential scanning calorimetry (DSC) (NETZSCH Gerätebau GmbH, Bavaria, Germany), powder X-ray diffraction (PXRD) (Rigaku Corporation, Tokyo, Japan), hot-stage polarized light microscopy (PLM) (Ningbo ShunYu Analytical Instrument Co., Ltd., Yuyao, China), near-infrared spectrum (NIR) spectroscopy (Thermo Fisher Scientific, Loughborough, UK), three-point bend testing, equilibrium solubility measurement, rheological characterization, in vitro drug release testing, release-kinetic analysis of printed tablets, and high-performance liquid chromatography (HPLC) (Agilent Technologies, Santa Clara, CA, USA).

## 3. Results and Discussion

### 3.1. Solid State Analysis of Raw Materials and Physical Mixtures

Thermal stability is a prerequisite for HME and FDM 3D printing because the drug and excipients must withstand the processing temperatures without degradation [38]. The thermal and structural properties of LY-11h and the selected polymers were therefore evaluated using TGA, DSC, PXRD, and PLM.

**Figure 2:**
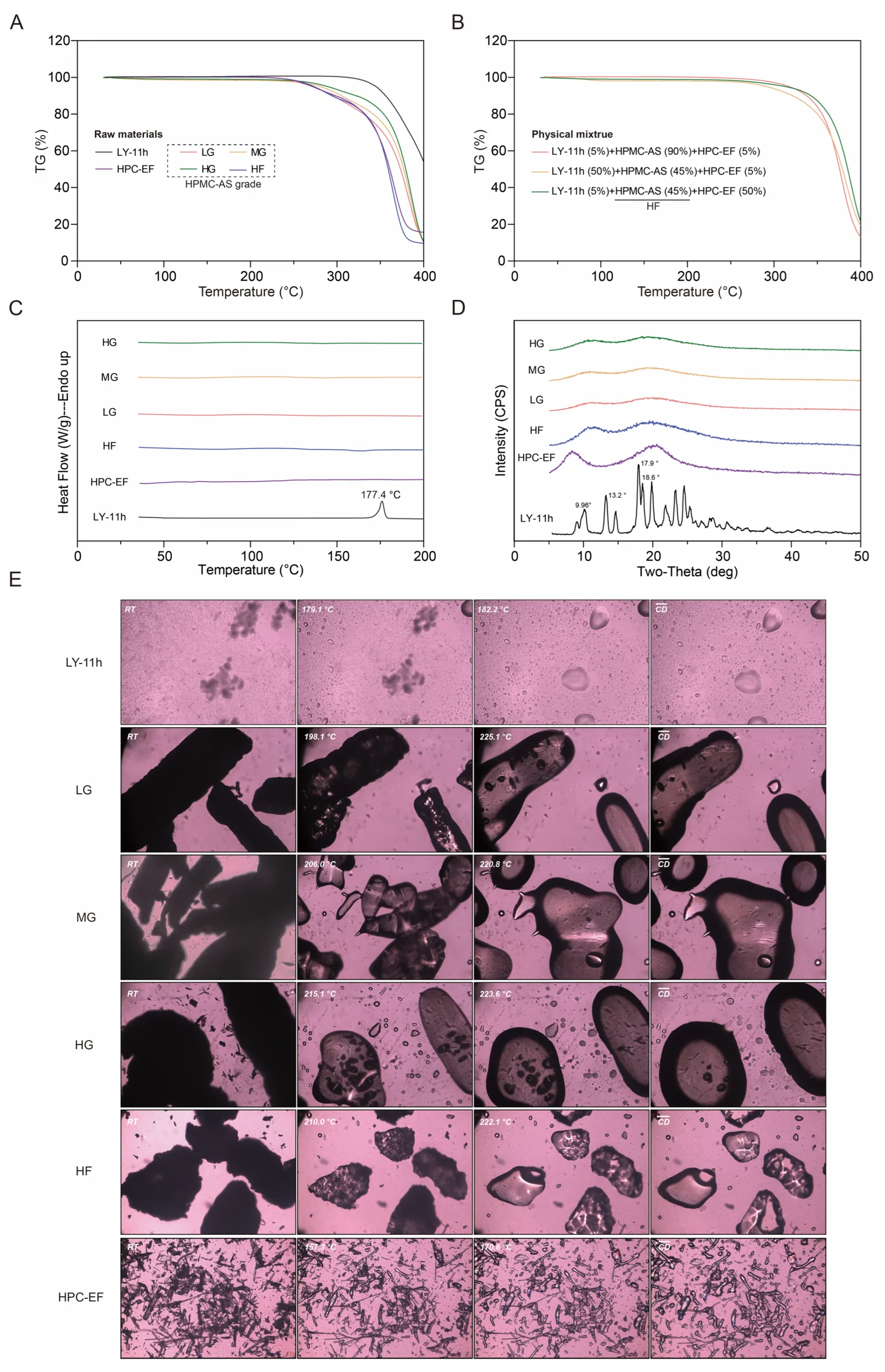
Physicochemical characterization of raw materials and physical mixtures. (**A**) TGA profiles of the raw materials, including LY-11h, HPC-EF, and different grades of HPMC-AS (LG, MG, HG, HF); (**B**) TGA profiles of physical mixtures composed of LY-11h, HPMC-AS HF, and HPC-EF at varying ratios; (**C**) DSC curves of the raw materials; (**D**) PXRD patterns of the raw materials; (**E**) PLM images of the raw materials at room temperature (RT) and during heating and cool down (CD). Scale bar = 100 μm.

As shown in Figure 2A, pure LY-11h exhibited good thermal stability, with no substantial mass loss below 300 °C, indicating the absence of gross volatile decomposition during the applied dynamic TGA heating program. The different HPMC-AS grades and HPC-EF showed major thermal degradation at higher temperatures, generally above the HME processing range. The physical mixtures of LY-11h, HPMC-AS, and HPC-EF showed degradation profiles similar to those of the individual components (Figure 2B). However, the absence of pronounced mass loss should not be interpreted as definitive evidence of complete chemical stability of HPMC-AS during high-temperature processing. Previous studies have demonstrated that the acetate and succinate substituents of HPMC-AS may undergo processing-dependent cleavage, resulting in the formation of free acetic and succinic acids [39,40,41]. The extent of this transformation depends on the polymer grade and the applied temperature, shear, and residence-time conditions. Such changes may precede pronounced bulk mass loss and cannot be adequately evaluated by conventional TGA alone. Nevertheless, the present results provided no evidence of severe thermal deterioration under the selected processing conditions. Although the FDM nozzle was set at 210 °C, the material experienced this temperature only during its brief passage through the heated nozzle rather than during prolonged isothermal exposure. In addition, the filaments were successfully printed, and the resulting tablets retained the intended pH-responsive release behavior, suggesting that severe or functionally significant degradation of the HPMC-AS matrix was unlikely. Future studies will incorporate GPC/SEC analysis, quantitative determination of free acetic and succinic acids, and drug-impurity profiling to further evaluate potential de-esterification, free-acid formation, and changes in the molecular-weight distribution of HPMC-AS after FDM processing.

The DSC curves showed a clear endothermic peak for crystalline LY-11h at approximately 177.4 °C, corresponding to its melting point (Figure 2C). In contrast, the HPMC-AS did not show sharp melting endotherms, consistent with their predominantly amorphous nature. PXRD patterns further confirmed the crystalline nature of LY-11h, which exhibited characteristic diffraction peaks, whereas HPMC-AS showed broad halo patterns (Figure 2D). HPC-EF displayed weak ordered-structure signals, indicating partial structural ordering rather than a fully amorphous state. PLM observations supported these findings: LY-11h appeared as crystalline particles that melted during heating, while HPMC-AS grades mainly underwent progressive softening and morphological changes without distinct crystalline melting events (Figure 2E). HPC-EF showed changes in its fibrous or ordered morphology during heating. These results indicate that LY-11h and the selected polymeric carriers possess sufficient thermal stability for HME and FDM processing, providing a necessary foundation for developing a thermally robust ASD strategy to overcome the poor oral formulation properties of LY-11h.

### 3.2. Formulation Study and Process Development of Drug-Loaded Filaments

#### 3.2.1. Rheological Studies

The formation of continuous and uniform filaments during HME is essential for successful FDM printing [42]. The melt must exhibit an appropriate balance between plasticity, which enables extrusion and shaping, and elasticity, which maintains filament dimensional stability after extrusion [43]. Rheological characterization was therefore performed to evaluate the viscoelastic behavior of formulations under the formulation conditions subsequently investigated in the single-factor studies and identify a preliminary thermal-processing region. Because the rheological measurements were conducted before the single-factor extrusion experiments, they were used as a preliminary assessment of thermal softening, melt-flow resistance, and the potential suitability of the formulations for HME and subsequent FDM printing. The complex viscosity (η*) values obtained under small-amplitude oscillatory conditions at a fixed frequency of 1 Hz were regarded as comparative indicators rather than as direct measurements of the shear viscosity inside the extruder or printer nozzle.

**Figure 3:**
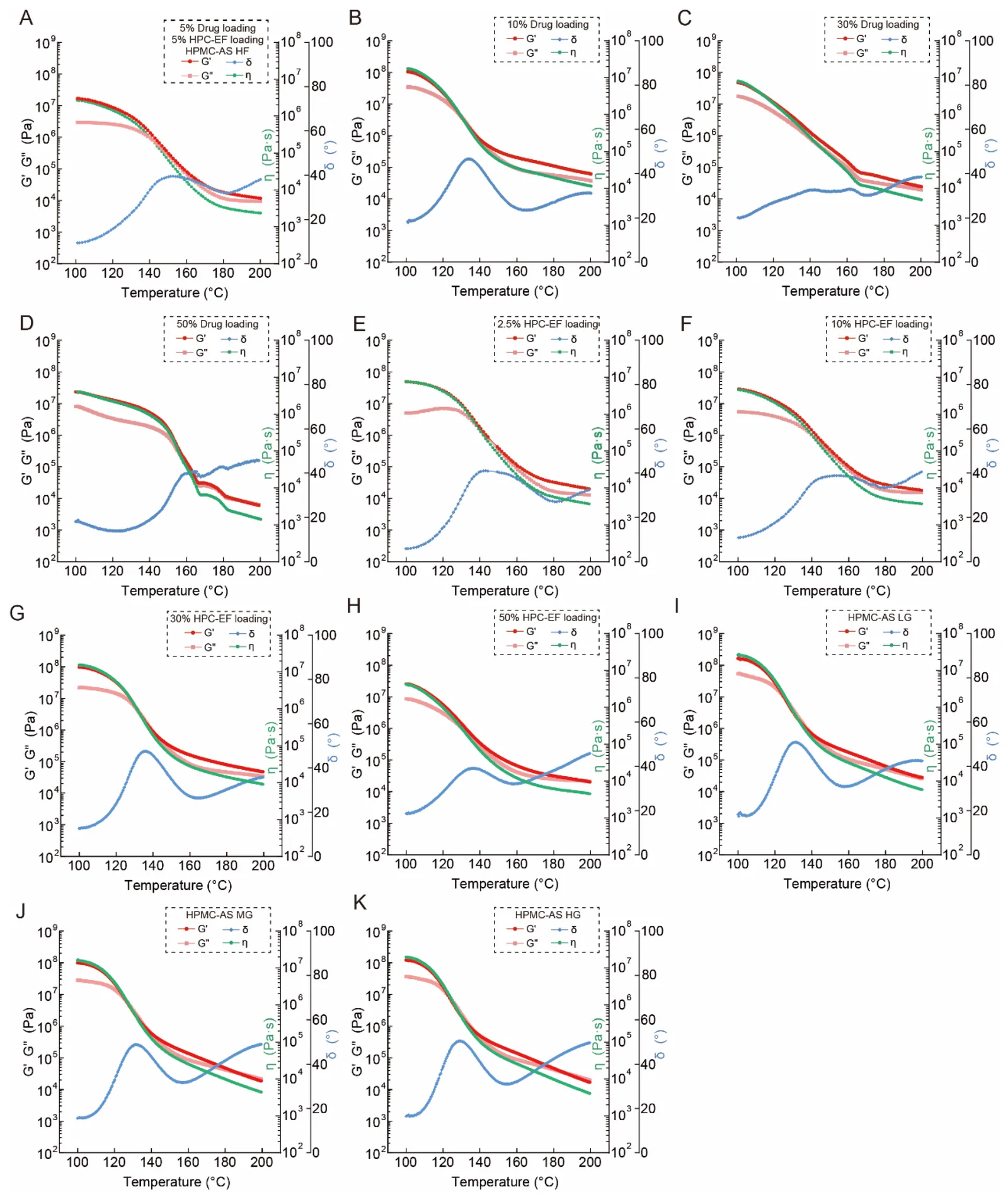
Rheological characterization of physical mixtures used in the single-factor screening studies. (**A**–**D**) Formulations containing different LY-11h loadings of 5%, 10%, 30%, and 50% (*w*/*w*), respectively; (**A**,**E**–**H**) formulations containing different HPC-EF contents of 5%, 2.5%, 10%, 30%, and 50% (*w*/*w*), respectively; and (**A**,**I**–**K**) formulations containing HPMC-AS HF, LG, MG, and HG, respectively. Panel (**A**) represents the reference formulation containing 5% LY-11h, 5% HPC-EF, and HPMC-AS HF and was shared among the three single-factor comparisons.

In the drug-loading group, the storage modulus (G′), loss modulus (G″), and complex viscosity generally decreased with increasing temperature (Figure 3A–D), indicating progressive softening of the formulation during heating. At approximately 100–120 °C, the formulations exhibited relatively high η* values, generally on the order of 10^6^–10^7^ Pa·s, indicating substantial resistance to melt deformation and flow. A marked reduction in η* occurred mainly between approximately 130 and 160 °C, where the viscosity decreased to approximately 10^5^–10^6^ Pa·s. At higher temperatures, η* decreased further, indicating increased melt flowability. Drug loading altered the temperature-dependent rheological response, particularly near the melting/softening region of LY-11h. These changes were likely associated with the thermal transition of LY-11h and its influence on drug–polymer interactions and melt rigidity. However, no simple monotonic relationship was observed between drug loading and η*, suggesting that complex viscosity alone was insufficient to identify an optimal drug-loading level.

Changing the HPC-EF content produced broadly similar rheological profiles across the tested formulations (Figure 3E–H). Although differences were observed in the transition region and high-temperature viscosity, no consistent monotonic trend in G′, G″, or complex viscosity was observed with increasing HPC-EF content. The overlapping profiles indicated that the investigated HPC-EF concentrations had broadly comparable thermal-softening behavior and that none produced an extreme increase or decrease in melt-flow resistance that would immediately preclude HME processing. Nevertheless, the similarity among the profiles also indicated that HPC-EF content could not be selected solely on the basis of η*.

Formulations containing different HPMC-AS grades showed similar rheological trends (Figure 3I–K). Both modulus values and complex viscosity decreased with increasing temperature, and no major differences were observed among the polymer grades. These similar profiles demonstrated that the investigated HPMC-AS grades possessed comparable bulk thermal-softening and flow behavior under the tested conditions. Therefore, rheological analysis did not identify any grade with an obvious processing disadvantage, but it also could not independently distinguish the most suitable grade. Selection of the HPMC-AS grade consequently required complementary assessment of drug amorphization, pH-dependent solubility, extrusion continuity, filament mechanical performance, and printability.

The rheological characterization provided a mechanistic basis for interpreting the extrusion behavior evaluated in the subsequent HME studies. The temperature-dependent decrease in η* indicated progressive polymer softening and improved melt flowability, which would be expected to facilitate drug–polymer mixing, die flow, and continuous filament formation during subsequent extrusion. From a processing perspective, excessively high η* at lower temperatures may increase extrusion resistance, torque, and screw load and may limit drug–polymer mixing and melt homogenization. Conversely, although a reduction in η* facilitates conveying and passage through the extrusion die, excessively low η* at high temperatures may reduce melt strength and increase the risk of filament deformation or diameter fluctuation after die exit. Accordingly, an intermediate thermal-softening region, rather than the lowest achievable viscosity, was considered more appropriate for producing continuous and dimensionally stable filaments.

The same balance is relevant to FDM printing. Sufficiently low melt viscosity is required to promote continuous flow through the printing nozzle and reduce the risk of interrupted deposition, whereas adequate elastic and cohesive strength is required for the deposited strand to retain its geometry and support successive printed layers. The simultaneous reduction in η* and retention of viscoelastic characteristics therefore suggested that the investigated formulations could potentially achieve both nozzle flow and post-deposition shape retention within an appropriate temperature range. However, because the rheological measurements were performed using physical mixtures under oscillatory conditions, their relevance to FDM printing was interpreted qualitatively and subsequently verified through filament preparation and printing experiments.

No universal rheological cut-off value was assigned because acceptable η* values depend on formulation composition, equipment configuration, screw geometry, throughput, and applied shear conditions. Instead, the pronounced decrease in η* between approximately 130 and 160 °C was regarded as an empirical candidate thermal-processing region for the subsequent single-factor extrusion studies. Overall, the rheological study provided a preliminary understanding of how temperature, drug loading, HPC-EF content, and HPMC-AS grade could affect melt flow, extrusion resistance, filament formation, and FDM printability, while the final formulation and process selections were determined through the subsequent experimental evaluation.

#### 3.2.2. Solid-State and Mechanical Evaluations

##### Drug Loading

**Figure 4:**
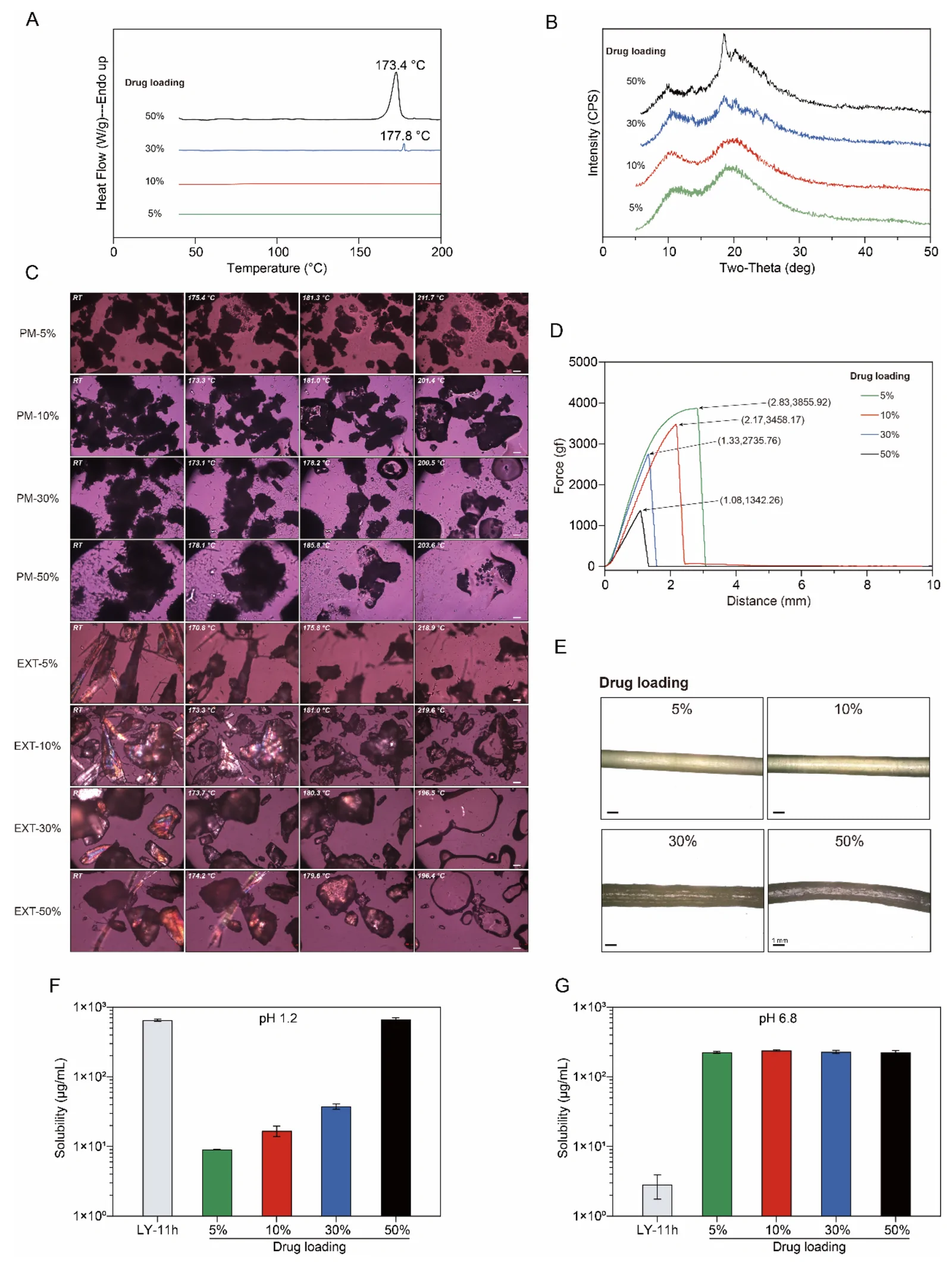
Effect of drug loading on the solid-state characteristics, mechanical properties, morphology, and equilibrium solubility of LY-11h-loaded formulations. (**A**) DSC thermograms and (**B**) PXRD patterns of extrudates containing 5%, 10%, 30%, and 50% (*w*/*w*) LY-11h. (**C**) Hot-stage polarized light microscopy images of the corresponding physical mixtures (PM) and extrudates (EXT) at room temperature (RT) and selected temperatures during heating. Scale bar = 100 μm. (**D**) Representative force–distance curves obtained from three-point bend testing of the extruded filaments. The annotated values indicate the distance at break and maximum force, respectively. (**E**) Representative optical microscopy images of filaments prepared at different drug loadings. Scale bar = 1 mm. Equilibrium solubility of pure LY-11h and formulations containing different drug loadings in media at (**F**) pH 1.2 and (**G**) pH 6.8. Data are presented as mean ± SD (n = 3). All formulations were extruded at 140 °C and 100 rpm.

DSC (Figure 4A) and PXRD (Figure 4B) analyses, as well as PLM (Figure 4C), showed that filaments containing 5% and 10% LY-11h were fully amorphous, with no detectable melting endotherms or crystalline diffraction peaks. At 30% drug loading, weak melting and diffraction signals appeared, indicating the onset of residual drug crystallinity. At 50% drug loading, distinct melting peaks and sharp diffraction signals confirmed substantial recrystallization.

Three-point bend testing showed an inverse relationship between drug loading and filament toughness (Figure 4D). The 5% drug-loading filament exhibited the highest maximum force and distance at break. Increasing drug loading reduced both parameters, and the 50% drug-loading filament became brittle and prone to breakage during printing. Optical microscopy (Figure 4E) further showed that high drug loading increased surface roughness and structural defects, which could compromise the dimensional stability of printed tablets.

Drug loading also strongly affected solubility and mechanical behavior (Figure 4F,G). The equilibrium solubility of pure LY-11h was 651.2 ± 25.3 μg/mL at pH 1.2 and only 2.8 ± 1.1 μg/mL at pH 6.8. At pH 1.2, the formulations containing 5%, 10%, and 30% LY-11h exhibited substantially reduced solubilities of 9.0 ± 0.1, 16.8 ± 2.8, and 37.5 ± 3.3 μg/mL, respectively, consistent with the limited dissolution of HPMC-AS under acidic conditions. In contrast, the 50% drug-loading formulation showed a solubility of 662.4 ± 40.8 μg/mL, comparable to that of pure LY-11h, which was consistent with the substantial residual crystallinity observed by DSC and PXRD. At pH 6.8, all drug-loading formulations exhibited markedly increased LY-11h solubility, ranging from 224.1 ± 7.1 to 239.1 ± 7.2 μg/mL, corresponding to approximately 80.0- to 85.4-fold enhancement relative to pure LY-11h. These results demonstrate that excessive LY-11h loading may compromise both amorphization and filament toughness, highlighting the need to balance dose strength with ASD stability and printability when developing LY-11h formulations for individualized AML therapy.

##### Moderate HPC-EF Loading Improves Filament Flexibility and Mechanical Robustness

**Figure 5:**
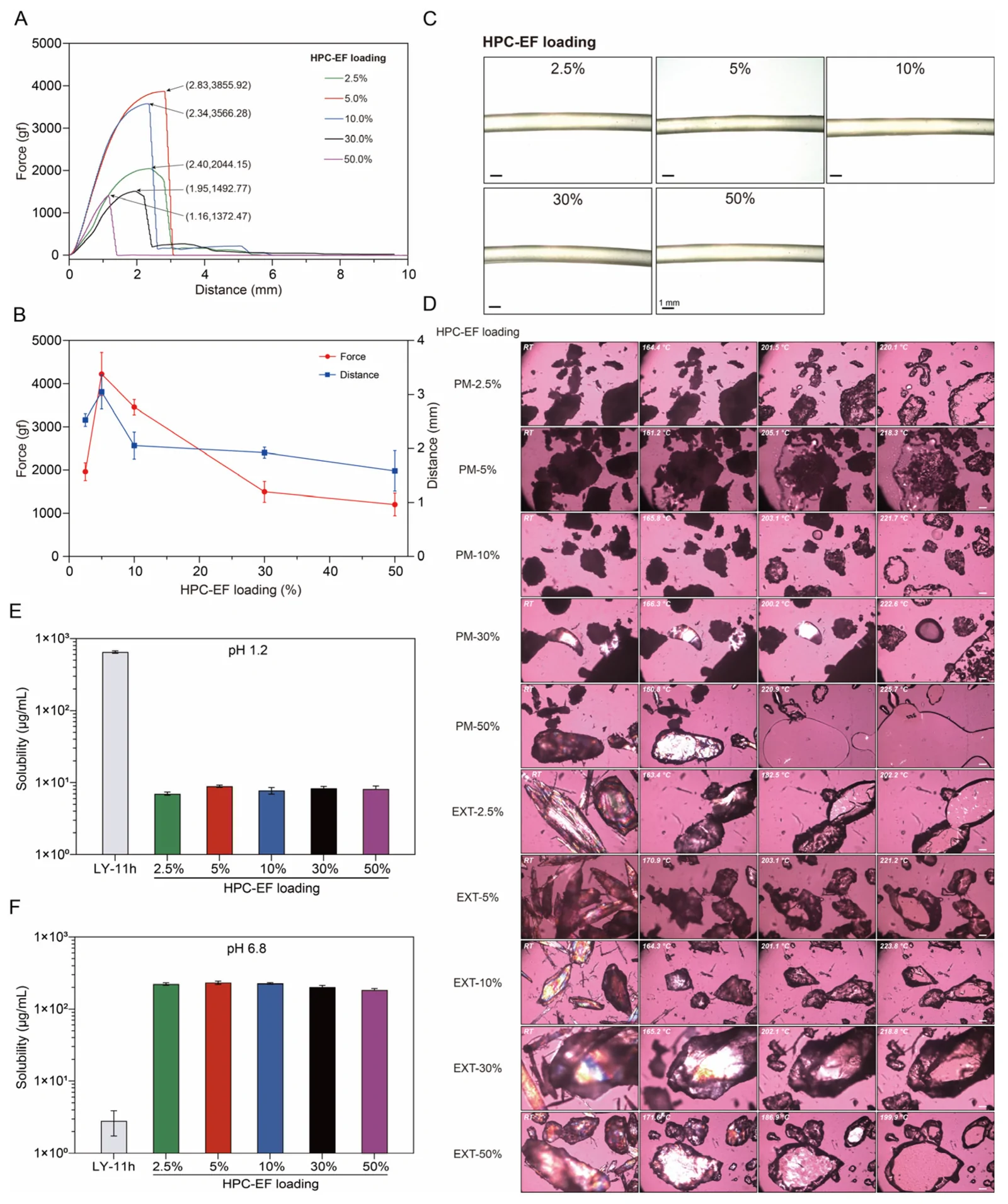
Effect of HPC-EF content on the mechanical properties, morphology, solid-state characteristics, and equilibrium solubility of LY-11h-loaded formulations. (**A**) Representative force–distance curves obtained from three-point bend testing of extruded filaments containing 2.5%, 5%, 10%, 30%, and 50% (*w*/*w*) HPC-EF. The annotated values indicate the distance at break and maximum force, respectively. (**B**) Effects of HPC-EF content on filament maximum force and distance at break. (**C**) Representative optical microscopy images of filaments prepared with different HPC-EF contents. Scale bar = 1 mm. (**D**) Hot-stage polarized light microscopy images of the corresponding physical mixtures (PM) and extrudates (EXT) at room temperature (RT) and selected temperatures during heating. Scale bar = 100 μm. Equilibrium solubility of pure LY-11h and formulations containing different HPC-EF contents in media at (**E**) pH 1.2 and (**F**) pH 6.8. Data are presented as mean ± SD (n = 3).

HPC-EF content significantly affected filament mechanical performance (Figure 5A,B). At low to moderate levels, increasing HPC-EF improved the maximum force and distance at break, with the 5% HPC-EF formulation showing the best overall performance. These results suggest that an appropriate amount of HPC-EF acts as a plasticizing component, improving melt flexibility during HME and enhancing filament toughness. Optical microscopy further showed that all HPC-EF-containing filaments had smooth and defect-free surfaces (Figure 5C), indicating good extrudability across the tested range.

When the HPC-EF content exceeded 10%, mechanical performance declined markedly. The 50% HPC-EF filament showed the lowest force and reduced elasticity. PLM (Figure 5D) confirmed that HPC-EF-containing formulations softened progressively during heating without crystalline melting events. Equilibrium-solubility testing showed that all HPC-EF-containing formulations maintained pronounced pH-dependent behavior (Figure 5E,F). At pH 1.2, their solubilities remained low, ranging from 7.0 ± 0.3 to 8.9 ± 0.3 μg/mL. At pH 6.8, the equilibrium solubility ranged from 183.5 ± 9.4 to 232.3 ± 11.6 μg/mL, representing approximately 65.5- to 83.0-fold enhancement relative to pure LY-11h. Although increasing the HPC-EF content to 30% and 50% moderately reduced the pH 6.8 solubility, all formulations retained substantial intestinal-solubility enhancement. This indicates that its main role was to modify filament mechanical properties rather than dissolution behavior. Therefore, HPC-EF mainly contributed to improving filament flexibility and mechanical robustness, helping to address the processability challenge of LY-11h ASD filaments and supporting the stable fabrication of FDM 3D-printed dosage forms for precision dosing.

Taken together, the rheological and mechanical results indicate that the selection of 5% HPC-EF was based primarily on its overall filament performance rather than on a distinct rheological minimum. The formulations containing different HPC-EF levels showed broadly overlapping viscoelastic profiles, with no consistent monotonic relationship between HPC-EF content and complex viscosity. Rheological screening alone therefore did not identify a clear optimal concentration. In contrast, the 5% HPC-EF formulation exhibited the most favorable combination of maximum force and distance at break among the tested formulations. This result suggests that a moderate HPC-EF content may provide a more favorable balance between filament flexibility and mechanical integrity than either lower or higher concentrations. One possible explanation is that moderate incorporation of HPC-EF facilitates deformation of the polymer matrix without excessively reducing its structural cohesion; however, polymer-chain mobility and interpolymer interactions were not directly measured in the present study. In addition, because extrusion torque was not systematically compared across the different HPC-EF concentrations, no conclusion can be drawn regarding whether 5% HPC-EF minimized torque or prevented torque-related processing failure. Accordingly, 5% HPC-EF was selected on the basis of the combined mechanical, morphological, and extrusion observations within the investigated formulation range.

##### HPMC-AS/HPC Dual Polymeric Matrix Combination

**Figure 6:**
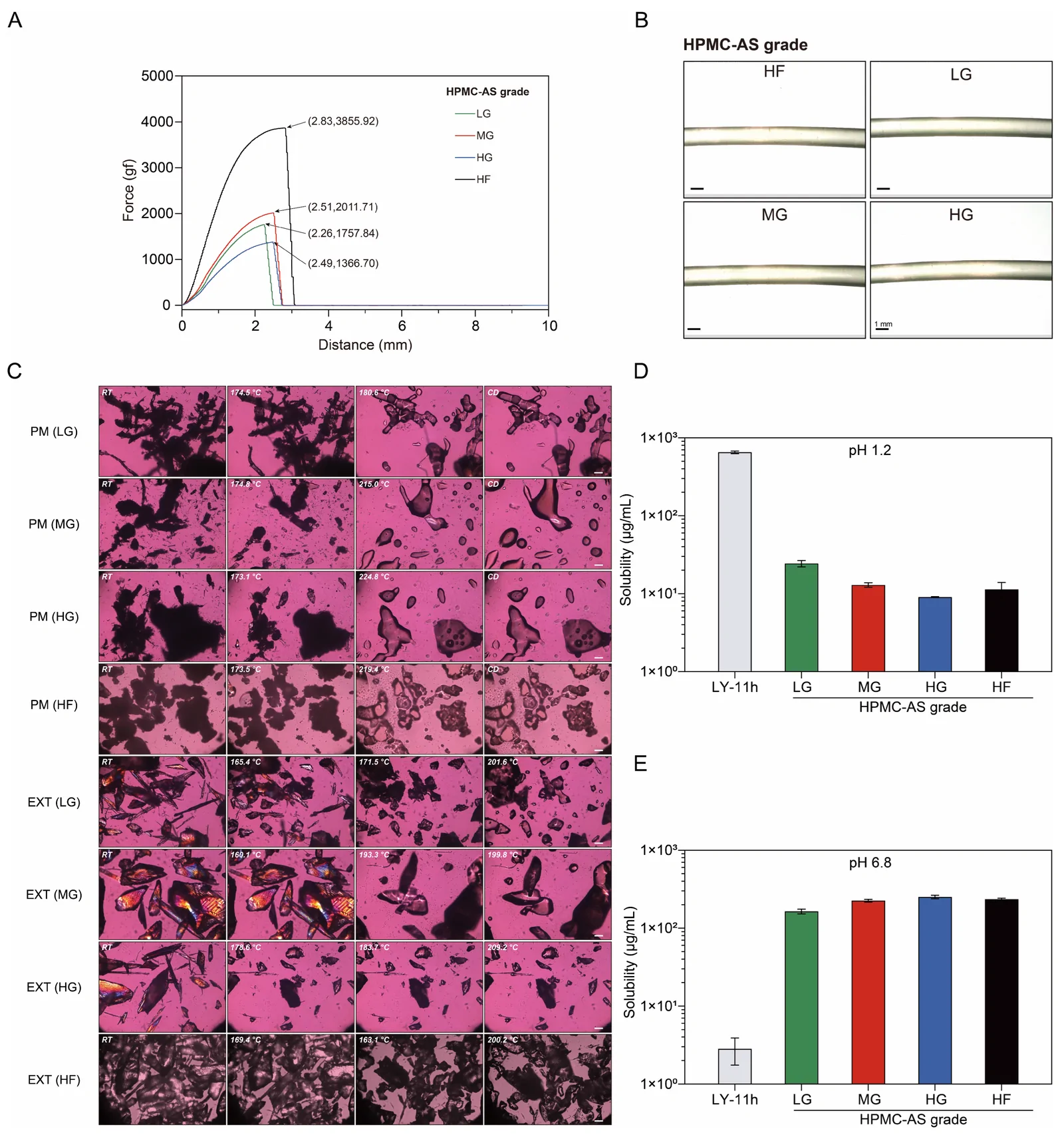
Effect of HPMC-AS grade on the mechanical properties, morphology, solid-state characteristics, and equilibrium solubility of LY-11h-loaded formulations. (**A**) Representative force–distance curves obtained from three-point bend testing of extruded filaments prepared using HPMC-AS grades LG, MG, HG, and HF. The annotated values indicate the distance at break and maximum force, respectively. (**B**) Representative optical microscopy images of filaments prepared with different HPMC-AS grades. Scale bar = 1 mm. (**C**) Hot-stage polarized light microscopy images of the corresponding physical mixtures (PM) and extrudates (EXT) at room temperature (RT), selected temperatures during heating, and after cooling down (CD). Scale bar = 100 μm. Equilibrium solubility of pure LY-11h and formulations containing different HPMC-AS grades in media at (**D**) pH 1.2 and (**E**) pH 6.8. Data are presented as mean ± SD (n = 3).

HPMC-AS grade significantly affected filament mechanical performance (Figure 6A). Filaments prepared with HPMC-AS HF exhibited the highest maximum force and distance at break, indicating excellent structural integrity suitable for continuous HME and FDM processing. In contrast, the LG grade showed the lowest mechanical strength, whereas the MG and HG grades exhibited intermediate performance. Optical microscopy further showed that all HPMC-AS-based filaments had uniform and defect-free surfaces (Figure 6B), suggesting good extrudability regardless of polymer grade.

PLM confirmed that all formulations remained free of distinct crystalline melting behavior during heating, with no observable API crystal domains in the extruded samples (Figure 6C). Solubility testing further showed that all HPMC-AS grades improved LY-11h dissolution in a pH-dependent manner (Figure 6D,E). At pH 1.2, the equilibrium solubilities of formulations containing HF, LG, MG, and HG were 11.4 ± 2.6, 24.4 ± 2.3, 12.9 ± 0.9, and 9.0 ± 0.2 μg/mL, respectively, all substantially lower than that of pure LY-11h. At pH 6.8, the corresponding values were 235.8 ± 7.7, 164.1 ± 11.9, 225.9 ± 9.6, and 252.7 ± 12.4 μg/mL, respectively, corresponding to approximately 58.6- to 90.2-fold enhancement relative to pure LY-11h. Although the magnitude of solubility enhancement varied among the grades, all HPMC-AS formulations substantially increased LY-11h solubility at pH 6.8. These findings indicate that HPMC-AS HF provided a favorable balance between mechanical strength, amorphous-state maintenance, and pH-responsive dissolution enhancement, making it suitable for addressing the limited intestinal solubility and oral delivery challenges of LY-11h.

Although the nominal dissolution thresholds of the tested HPMC-AS grades differed, all formulations showed enhanced LY-11h solubility in the pH 6.8 medium because this pH was above or close to the dissolution threshold of each tested polymer type. Consequently, the final grade selection was primarily determined by the combined solid-state, mechanical, and processing performance rather than by equilibrium solubility at pH 6.8 alone.

### 3.3. Preliminary Investigation of Filament Preparation via HME Processing

#### 3.3.1. Processing Temperature

**Figure 7:**
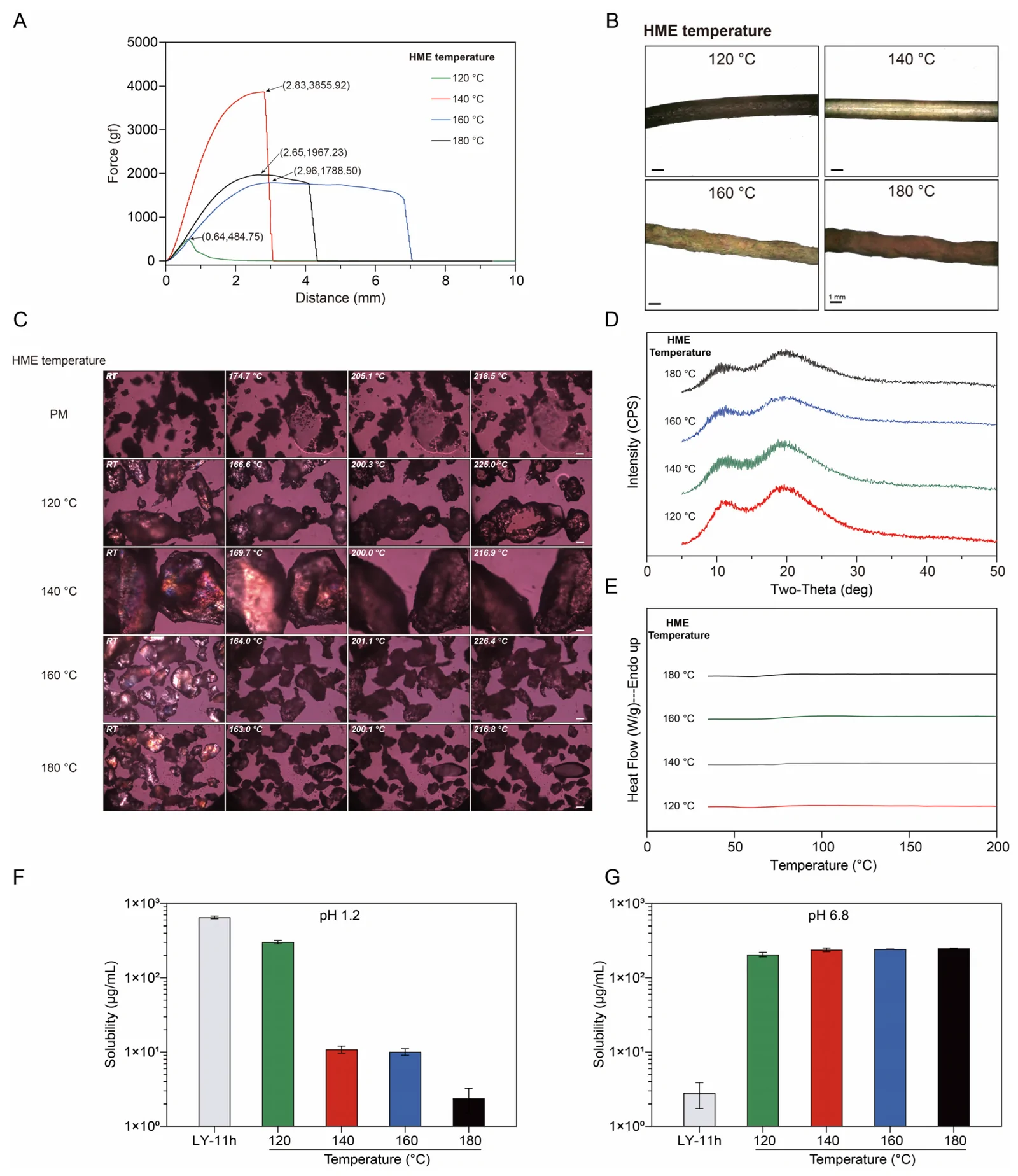
Effect of HME temperature on the mechanical properties, morphology, solid-state characteristics, and equilibrium solubility of LY-11h-loaded formulations. (**A**) Representative force–distance curves obtained from three-point bend testing of filaments extruded at 120, 140, 160, and 180 °C. The annotated values indicate the distance at break and maximum force, respectively. (**B**) Representative optical microscopy images of filaments prepared at different extrusion temperatures. Scale bar = 1 mm. (**C**) Hot-stage polarized light microscopy images of the physical mixture (PM) and extrudates prepared at different HME temperatures, recorded at room temperature (RT) and selected temperatures during heating. Scale bar = 100 μm. (**D**) PXRD patterns and (**E**) DSC thermograms of extrudates prepared at different HME temperatures. Equilibrium solubility of pure LY-11h and formulations extruded at different temperatures in media at (**F**) pH 1.2 and (**G**) pH 6.8. Data are presented as mean ± SD (n = 3). All formulations contained 5% (*w*/*w*) LY-11h and were extruded at a screw speed of 100 rpm.

Extrusion temperature significantly affected filament mechanical performance and morphology (Figure 7A,B). Filaments extruded at 140 °C exhibited the highest maximum force and distance at break, together with a smooth and uniform surface. At 120 °C, the filament showed reduced mechanical strength and increased brittleness, with a maximum force of approximately 485 gf, and appeared white and opaque under optical microscopy. In contrast, temperatures above 160 °C caused filament discoloration, surface defects, and reduced mechanical integrity.

Hot-stage PLM supported the absence of crystalline melting events in all extruded samples (Figure 7C). PXRD and DSC analysis confirmed that all formulations remained amorphous, showing only broad halos without distinct crystalline diffraction peaks regardless of extrusion temperature (Figure 7D,E). Solubility testing showed that all ASD formulations markedly enhanced LY-11h solubility at pH 6.8 compared with the pure drug, whereas solubility at pH 1.2 gradually decreased with increasing extrusion temperature (Figure 7F,G). The pH 6.8 equilibrium solubility increased from 205.9 ± 14.4 μg/mL at 120 °C to 238.6 ± 13.3, 244.4 ± 0.9, and 250.4 ± 1.3 μg/mL at 140, 160, and 180 °C, respectively. In parallel, the pH 1.2 solubility decreased markedly from 303.9 ± 16.6 μg/mL at 120 °C to 10.9 ± 1.2, 10.1 ± 1.0, and 2.4 ± 0.9 μg/mL at 140, 160, and 180 °C, respectively. These results suggest that extrusion temperature must be carefully optimized to promote LY-11h amorphization and melt homogeneity while avoiding thermal or mechanical deterioration, which is critical for producing ASD filaments capable of improving intestinal dissolution and downstream printability.

#### 3.3.2. Mixing Sections and Screw Configurations

**Figure 8:**
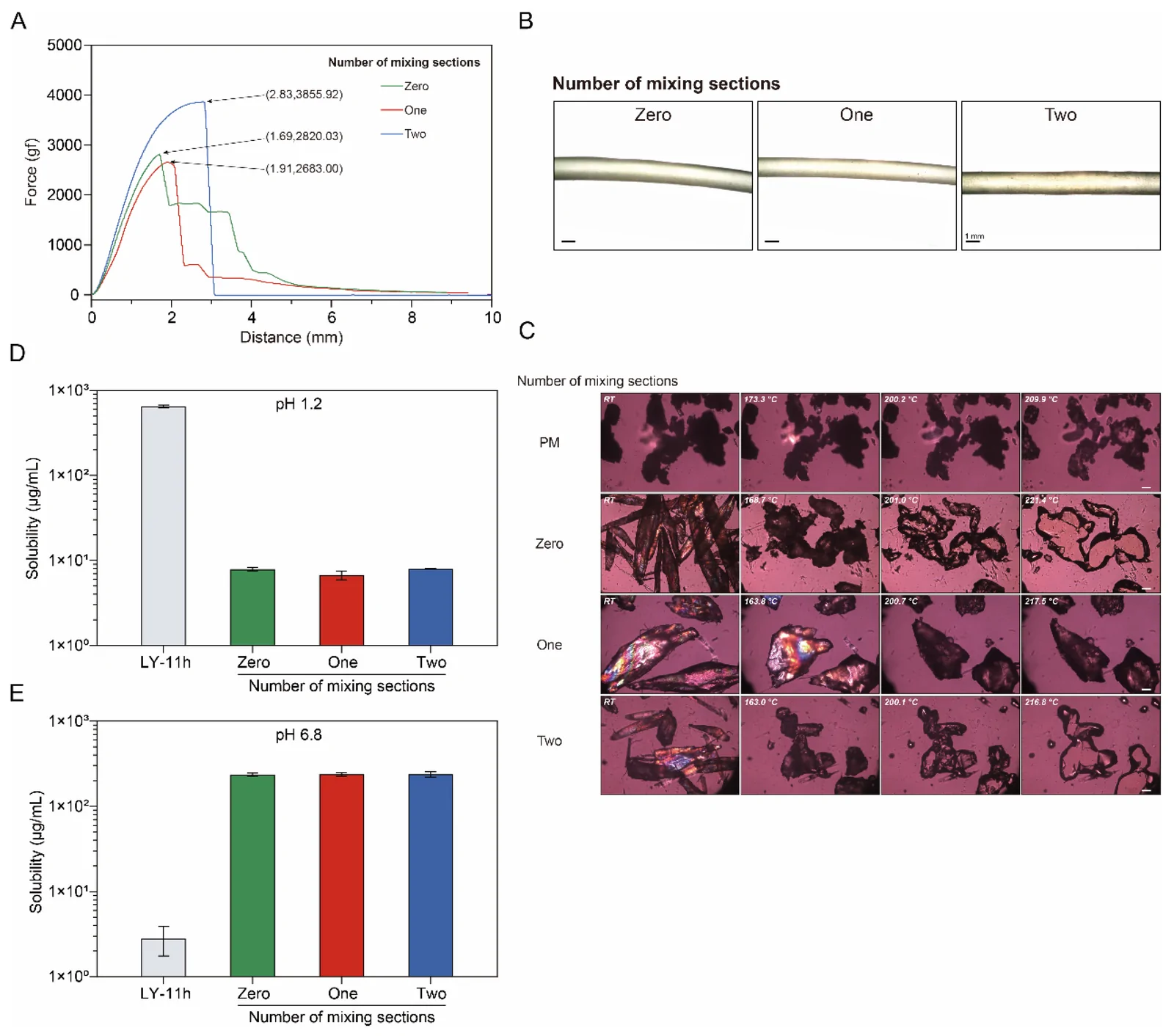
Effect of the number of mixing sections on the mechanical properties, morphology, solid-state characteristics, and equilibrium solubility of LY-11h-loaded formulations. (**A**) Representative force–distance curves obtained from three-point bend testing of filaments prepared using screw configurations containing zero, one, or two mixing sections. The annotated values indicate the distance at break and maximum force, respectively. (**B**) Representative optical microscopy images of the corresponding extruded filaments. Scale bar = 1 mm. (**C**) Hot-stage polarized light microscopy images of the physical mixture (PM) and extrudates prepared using zero, one, or two mixing sections, recorded at room temperature (RT) and selected temperatures during heating. Scale bar = 100 μm. Equilibrium solubility of pure LY-11h and formulations prepared with different numbers of mixing sections in media at (**D**) pH 1.2 and (**E**) pH 6.8. Data are presented as mean ± SD (n = 3).

The number of mixing sections directly affected filament mechanical performance (Figure 8A). Increasing the number of mixing sections from zero to two improved both the maximum force and distance at break, with the two-section configuration showing the best overall performance. Filaments prepared without mixing sections exhibited lower strength and an irregular fracture pattern. Optical microscopy showed that all filaments had smooth surfaces (Figure 8B), indicating that additional mixing sections improved melt uniformity and mechanical integrity without inducing obvious surface defects.

PLM analysis showed that all extruded formulations remained free of distinct crystalline melting events during heating, regardless of the number of mixing sections (Figure 8C). Solubility testing further demonstrated that all ASD formulations markedly enhanced LY-11h solubility at pH 6.8 compared with the pure drug, while maintaining low solubility at pH 1.2, with comparable performance across different mixing-section configurations (Figure 8D,E). The pH 1.2 solubilities were 7.9 ± 0.4, 6.7 ± 0.8, and 8.0 ± 0.1 μg/mL for zero, one, and two mixing sections, respectively, whereas the corresponding pH 6.8 values were 235.9 ± 10.1, 237.7 ± 13.0, and 238.2 ± 18.1 μg/mL. The improvement in filament performance with additional mixing sections indicates that enhanced drug–polymer homogenization is beneficial for stabilizing LY-11h in the amorphous state and supporting the preparation of mechanically reliable filaments for FDM-printed oral dosage forms designed for precision dosing.

#### 3.3.3. Moderate Screw Speed

**Figure 9:**
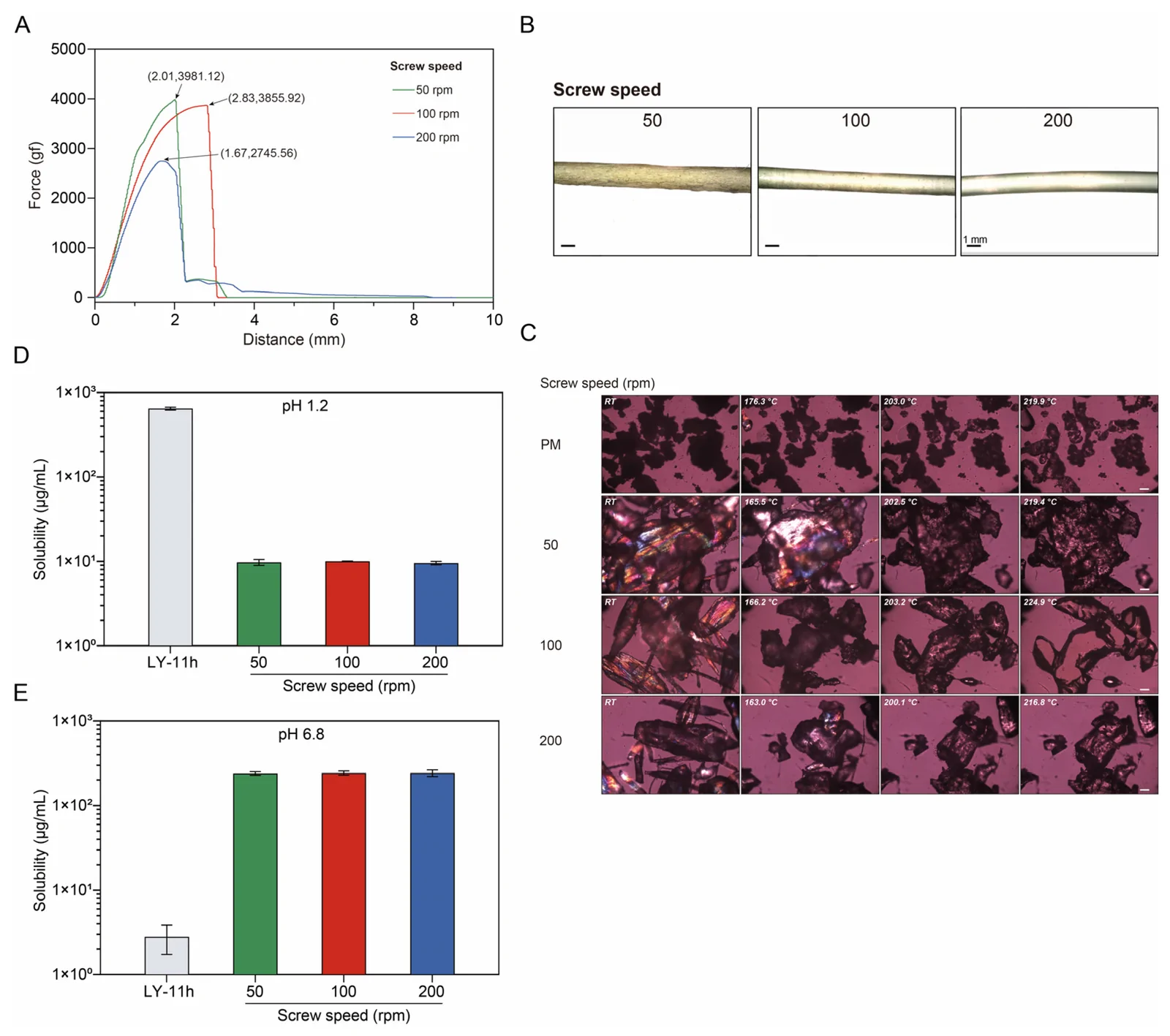
Effect of screw speed on the mechanical properties, morphology, solid-state characteristics, and equilibrium solubility of LY-11h-loaded formulations. (**A**) Representative force–distance curves obtained from three-point bend testing of filaments extruded at screw speeds of 50, 100, and 200 rpm. The annotated values indicate the distance at break and maximum force, respectively. (**B**) Representative optical microscopy images of filaments prepared at different screw speeds. Scale bar = 1 mm. (**C**) Hot-stage polarized light microscopy images of the physical mixture (PM) and extrudates prepared at 50, 100, and 200 rpm, recorded at room temperature (RT) and selected temperatures during heating. Scale bar = 100 μm. Equilibrium solubility of pure LY-11h and formulations prepared at different screw speeds in media at (**D**) pH 1.2 and (**E**) pH 6.8. Data are presented as mean ± SD (n = 3).

Screw speed affected filament mechanical performance (Figure 9A). The 100-rpm condition produced the highest maximum force, whereas the 50-rpm and 200-rpm conditions showed slightly reduced strength. At 50 rpm, the filament exhibited lower density and minor surface roughness, while excessive shear at 200 rpm reduced filament flexibility (Figure 9B). Nevertheless, all formulations showed adequate uniformity and mechanical strength for FDM 3D printing.

PLM analysis confirmed that all extruded formulations remained free of distinct crystalline melting events during heating, regardless of screw speed (Figure 9C). Solubility testing further showed that all ASD formulations markedly enhanced LY-11h solubility at pH 6.8 compared with the pure drug, while maintaining low solubility at pH 1.2, with comparable performance across different screw speeds (Figure 9D,E). At 50, 100, and 200 rpm, the pH 1.2 solubilities were 9.8 ± 0.8, 10.1 ± 0.1, and 9.6 ± 0.4 μg/mL, respectively, whereas the corresponding pH 6.8 values were 241.2 ± 11.9, 244.5 ± 14.5, and 243.8 ± 22.4 μg/mL. These findings show that a moderate screw speed can balance shear-induced mixing and filament integrity, thereby supporting the production of LY-11h ASD filaments with sufficient mechanical quality for FDM printing and flexible dose adjustment.

Overall, the single-factor studies established a practical formulation and processing window for subsequent multivariate optimization. HPMC-AS HF was selected because it provided the highest filament maximum force and distance at break while maintaining amorphous-state and pH-responsive dissolution performance. An HPC-EF content of 5% was selected because it provided the most favorable mechanical performance, whereas higher HPC-EF contents reduced filament strength. Two mixing sections were retained because they improved melt homogenization and filament mechanical integrity. These variables were therefore maintained as fixed conditions in the subsequent optimization.

In contrast, drug loading, extrusion temperature, and screw speed produced substantial changes in filament solid-state, mechanical, morphological, and processing properties and were therefore selected as the DoE factors. The 50% drug-loading formulation was excluded because of substantial residual crystallinity and brittleness, and 30% was retained as the upper practical boundary. Similarly, 180 °C was excluded because of filament discoloration, surface defects, and reduced mechanical integrity, while the 120–160 °C range was retained. The 50-rpm condition was excluded because it produced lower-density filaments with minor surface roughness, whereas 100–200 rpm represented a practically suitable operating range. Accordingly, the DoE levels were set at 5%, 17.5%, and 30% for drug loading; 120, 140, and 160 °C for extrusion temperature; and 100, 150, and 200 rpm for screw speed. The 30% drug-loading level was retained as the upper practical boundary at which weak residual crystallinity began to emerge, while amorphization was included as an explicit DoE response to account for solid-state changes within the investigated design space.

### 3.4. Response Surface Analysis and Optimization of LY-11h ASD Filament Manufacturing

#### 3.4.1. Critical Process Parameter Investigation via Box–Behnken Design

**Figure 10:**
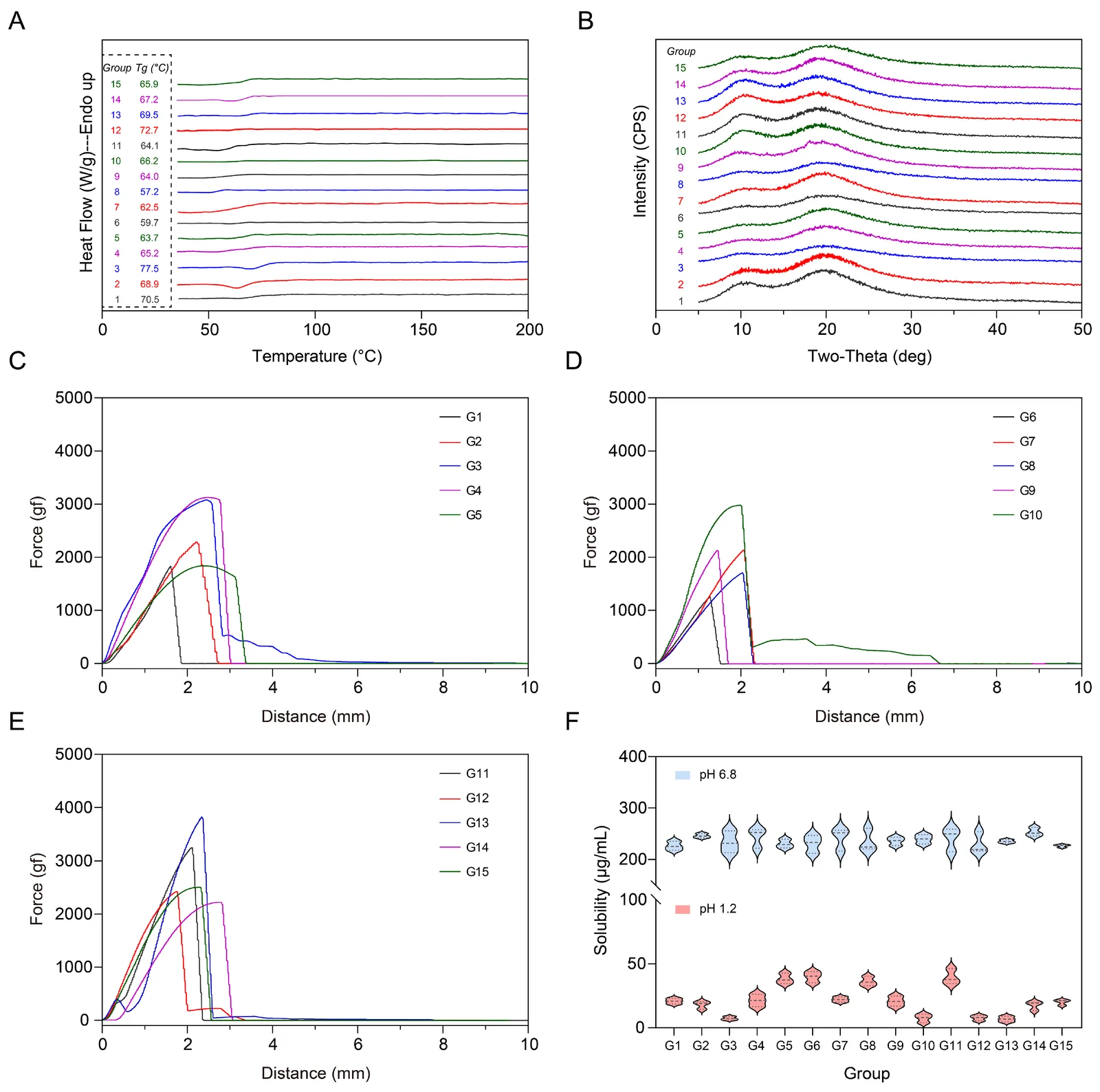
Characterization of 15 groups from the BBD. (**A**) DSC curves; (**B**) PXRD; (**C**–**E**) Three-point bend; (**F**) pH-dependent solubility profiles at pH 1.2 and pH 6.8.

A three-factor, three-level Box–Behnken design (BBD) was used to systematically investigate the combined effects of extrusion temperature, screw speed, and drug loading on the performance of 3D-printable LY-11h ASDs. The measured responses included filament mechanical properties, amorphization efficiency, glass-transition temperature (Tg), and extrusion torque, and the full experimental matrix is presented in Table S1. The design comprised 15 experimental runs, including three independently prepared center-point runs conducted at 140 °C, 150 rpm, and 17.5% (*w*/*w*) drug loading. Each response was measured in triplicate (n = 3), and the mean value was used for DoE modeling. DSC and PXRD analyses confirmed that most formulations formed single-phase amorphous systems, with Tg values ranging from 57.2 to 77.5 °C and broad halo patterns without distinct Bragg peaks (Figure 10A,B). Hot-stage PLM observations further supported the absence of detectable crystalline LY-11h in the BBD formulations (Figure S1). These results indicate strong drug–polymer interactions and a high degree of amorphization across the BBD formulations.

The mechanical performance of the 15 formulations showed substantial variability, as reflected by the three-point bend results (Figure 10C–E). Formulations prepared at moderate temperatures, intermediate screw speeds, and lower drug loadings generally exhibited higher maximum force and distance at break, indicating better filament flexibility and structural integrity for FDM printing. Solubility testing further showed that all ASD formulations markedly enhanced LY-11h solubility at pH 6.8 compared with the pure drug, while maintaining low solubility at pH 1.2, with no major differences among the 15 runs (Figure 10F). These results suggest that the investigated variables mainly influenced the physical and mechanical properties of the filaments rather than the dissolution enhancement of the amorphous drug.

The reproducibility of the BBD experiments was further evaluated using the three independently prepared center-point batches. The center-point batches showed mean ± SD values of 2.36 ± 0.15 mm for distance at break, 2678.68 ± 148.26 gf for maximum force, 96.42 ± 0.07% for amorphization, 66.10 ± 1.01 °C for Tg, and 3.93 ± 0.06 N·m for extrusion torque. The corresponding coefficients of variation were 6.50%, 5.53%, 0.08%, 1.54%, and 1.47%, respectively. The low variability among the independently prepared center-point batches demonstrated acceptable reproducibility of the HME process and the associated response measurements.

Overall, the BBD results indicate that extrusion temperature, screw speed, and drug loading mainly governed filament mechanical performance and printability, while the ASD system consistently maintained pH-dependent solubility enhancement, directly addressing both the poor intestinal dissolution and individualized dosing requirements of LY-11h.

#### 3.4.2. Response Surface Methodology for Process Optimization

**Figure 11:**
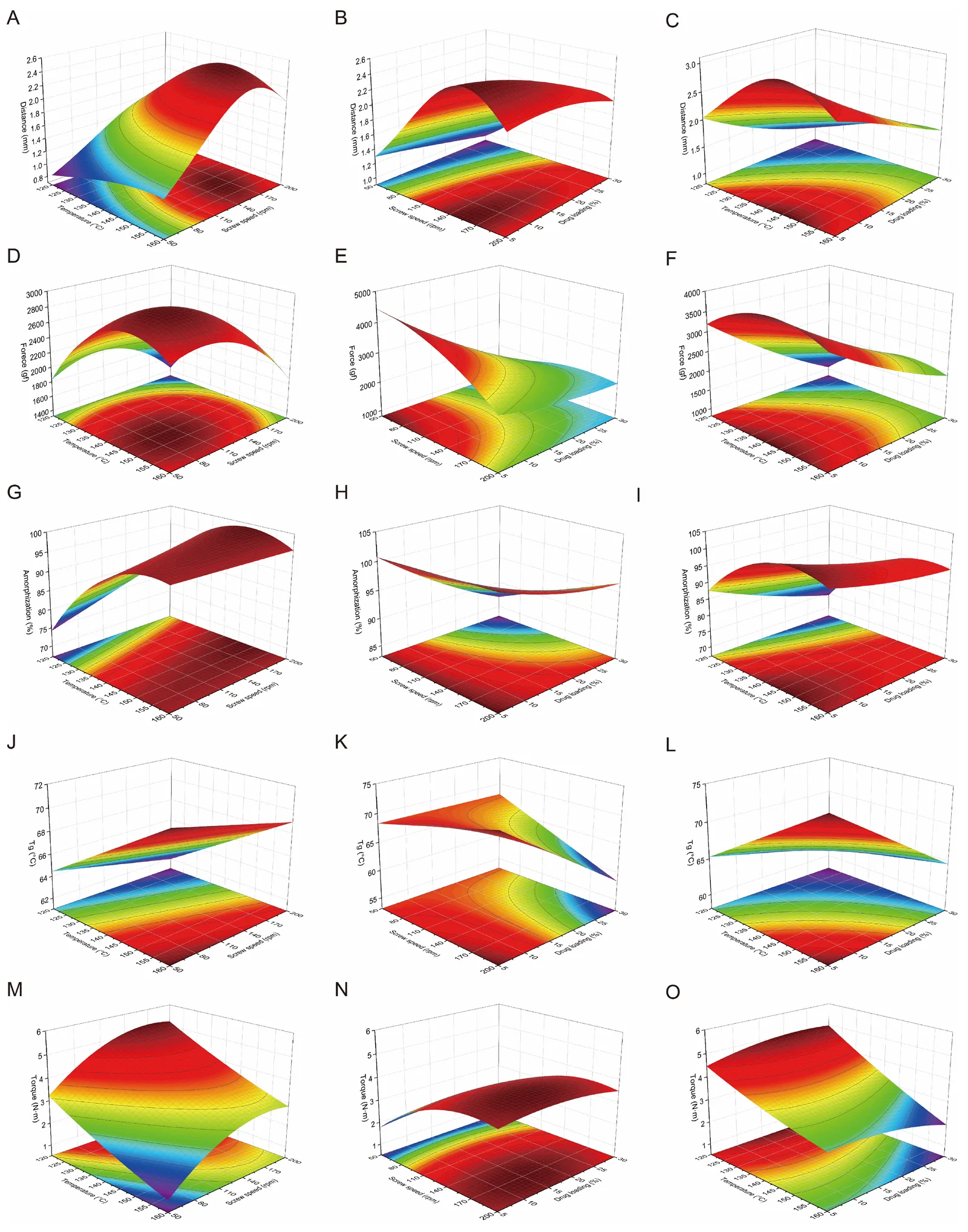
Response surface plots for pairwise factor interactions. (**A**–**C**) Distance; (**D**–**F**) Force; (**G**–**I**) Amorphization; (**J**–**L**) Tg; (**M**–**O**) Torque. The color gradient represents the magnitude of the predicted response, ranging from blue (lower values) through green/yellow (intermediate values) to red (higher values).

RSM was applied to the BBD dataset to evaluate the individual and interactive effects of extrusion temperature, screw speed, and drug loading on the critical quality attributes of LY-11h ASD filaments. The response surface plots visualized their effects on distance at break, maximum force, amorphization efficiency, Tg, and extrusion torque (Figure 11). Quadratic response-surface models were fitted after comparing different response transformations. An inverse transformation was selected for distance at break, with R^2^ = 0.8192 and an overall model *p*-value of 0.0016. The original response scale was retained for maximum force, with R^2^ = 0.8524 and *p* = 0.0045. A square-root transformation was selected for amorphization efficiency, with R^2^ = 0.8954 and *p* = 0.0002, whereas the original response scales were retained for Tg and extrusion torque, with R^2^ values of 0.9557 and 0.9155 and *p*-values of 0.0003 and 0.0046, respectively. All selected models were statistically significant based on ANOVA, using *p* < 0.05 as the significance criterion. The selected response transformations, model-fit statistics, and polynomial equations are summarized in the Supplementary Information.

Extrusion temperature, screw speed, and drug loading showed clear interactive effects on filament mechanical properties (Figure 11A–F). Based on the response-surface trends, extrusion temperature showed the clearest influence on distance at break, whereas maximum force was particularly sensitive to drug loading and its interactions with the processing conditions. For distance at break, moderate temperature and screw speed improved filament flexibility, whereas insufficient temperature resulted in reduced distance values (Figure 11A). The beneficial effect of temperature became less pronounced at higher drug loading, and lower distance values were observed when high drug loading was combined with unfavorable screw-speed conditions (Figure 11B,C). For maximum force, the highest values were generally obtained under moderate temperature and screw-speed conditions, while either insufficient melting or excessive shear led to reduced mechanical strength (Figure 11D). Drug loading further modulated the force response, particularly through its interactions with temperature and screw speed (Figure 11E,F). Thus, the mechanical responses depended on the balance among thermal softening, drug content, and shear conditions rather than on any single processing variable alone.

Amorphization efficiency was mainly governed by extrusion temperature and drug loading (Figure 11G–I). These two variables showed the most pronounced effects on amorphization efficiency within the investigated design space. Low temperature provided insufficient thermal energy for complete amorphization, especially at higher drug loading. Increasing extrusion temperature promoted drug amorphization, and this effect was particularly evident in formulations with higher drug content (Figure 11H). Screw speed also contributed to amorphization by regulating melt mixing and heat transfer, but it could not fully compensate for the negative effect of high drug loading under insufficient thermal conditions (Figure 11G,I).

Tg was influenced by the combined effects of drug loading and processing conditions (Figure 11J–L). Among the investigated variables, drug loading showed the clearest response-surface effect on Tg. Increasing drug loading generally reduced Tg, which may be attributed to the plasticizing effect of LY-11h within the polymer matrix (Figure 11K). Higher extrusion temperature appeared to moderate this Tg reduction, possibly by improving drug–polymer dispersion and single-phase homogeneity. The interaction between screw speed and drug loading was less pronounced, although appropriate shear may contribute to more uniform drug distribution and thereby slightly affect Tg (Figure 11L).

Extrusion torque was strongly affected by temperature-dependent melt viscosity and formulation composition (Figure 11M–O). Extrusion temperature showed the most pronounced influence on torque, whereas drug loading and screw speed contributed through formulation- and shear-dependent interactions. At lower extrusion temperatures, the polymer matrix exhibited higher viscosity, resulting in increased torque, especially at higher drug loading (Figure 11N). Increasing temperature reduced torque through thermal softening of the melt. The influence of screw speed was more evident under low-temperature conditions, where shear effects altered flow resistance, whereas torque became less sensitive to screw speed at higher temperatures because of improved melt fluidity (Figure 11M). The screw speed–drug loading interaction further reflected the combined effects of shear and formulation composition on extrusion resistance (Figure 11O).

No fixed numerical acceptance limits or independent desirability targets were assigned to the individual responses. Instead, the response-surface results were interpreted using directional evaluation criteria. Higher distance at break and maximum force were considered favorable because they supported filament flexibility, mechanical robustness, feeding stability, and resistance to brittle fracture. Amorphization efficiency was favored toward 100%, while comparatively higher Tg values within the investigated design space were considered favorable as an indicator of reduced molecular mobility. Extrusion torque was maintained within a stable operating range because excessively high torque may indicate inadequate melt flow and increased equipment load, whereas excessively low torque may reflect insufficient melt resistance or mixing. Therefore, the objective was to identify a practical formulation–processing region that balanced filament flexibility, mechanical strength, amorphization, Tg, and extrusion stability rather than to define a single mathematically optimized condition.

The RSM analysis clarified the formulation–process window required to obtain LY-11h ASD filaments with balanced amorphization, mechanical strength, and extrusion stability, providing a rational basis for overcoming the manufacturability limitations associated with LY-11h oral dosage development.

#### 3.4.3. Machine-Learning-Assisted Prediction of Filament Mechanical Performance and Formulation–Process Optimization

Following the DoE and RSM analyses, machine-learning models were further developed to predict the printability-related mechanical properties of LY-11h ASD filaments. Maximum force and distance at break were selected as prediction targets because they directly influence filament feeding stability, resistance to breakage, printing success rate, and dimensional consistency during FDM printing. Unlike conventional response-surface models, ML algorithms can capture nonlinear and non-monotonic formulation–process–property relationships, which may be particularly useful when multiple variables simultaneously affect melt behavior, drug–polymer dispersion, and filament structural integrity. Therefore, ML modeling was introduced as a complementary data-driven tool to predict filament mechanical performance from formulation and HME process variables and to support formulation–process optimization.

**Figure 12:**
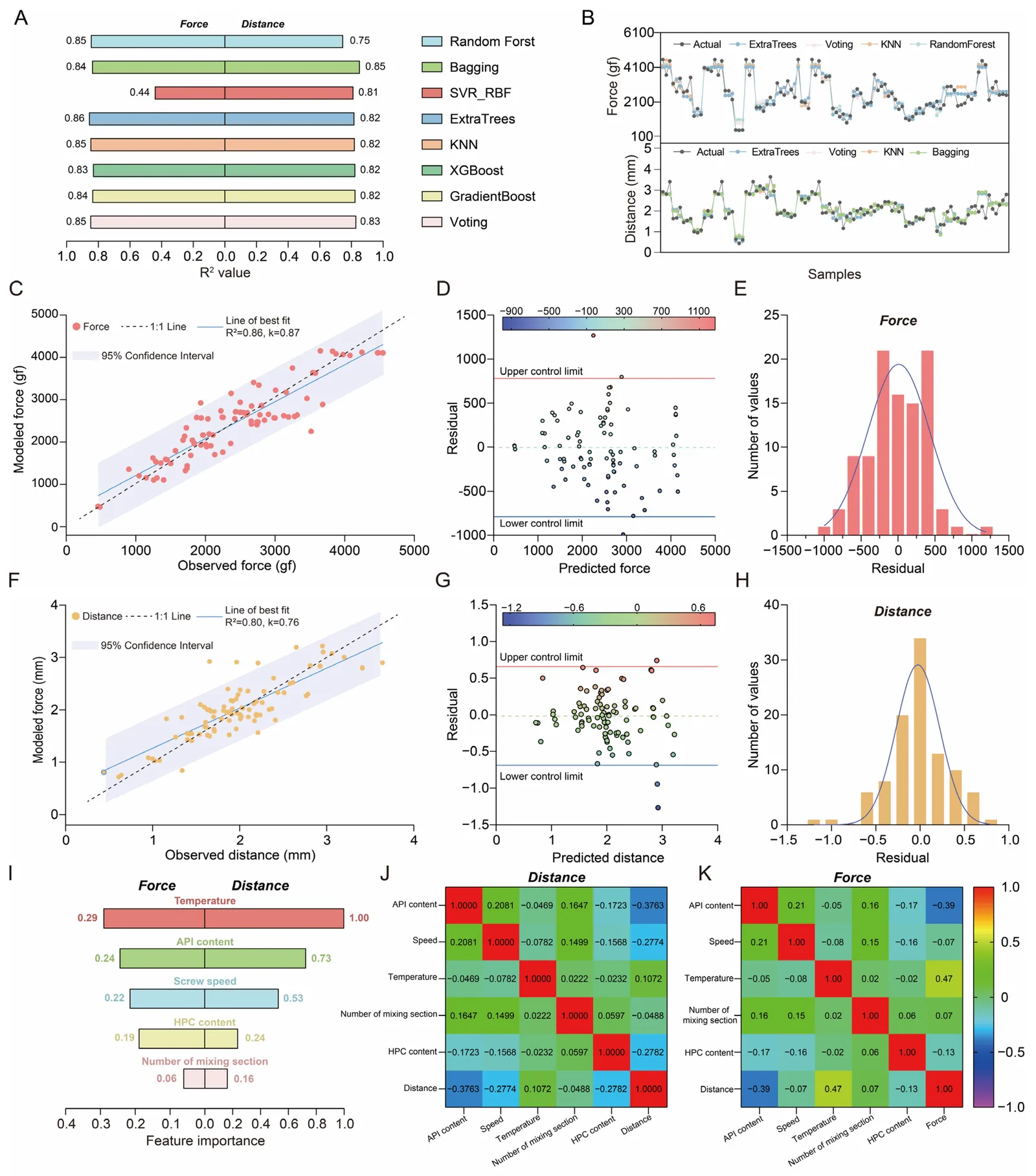
ML prediction of filament mechanical properties. (**A**) R^2^ values of different algorithms for force and distance prediction; (**B**) Validation of the top four high-performance ML algorithms; (**C**,**F**) Observed vs. predicted plots for force and distance; (**D**,**G**) Residual plots of the ExtraTrees (Force) and Bagging (Distance) model; (**E**,**H**) Histograms of residuals for force and distance; (**I**) Feature importance ranking for force and distance. (**J**,**K**) Correlation heatmaps of variables for distance and force. The black dashed diagonal lines in panels C and F represent the 1:1 reference lines, while the horizontal dashed lines in panels D and G represent the zero-residual reference lines.

Different ML algorithms were first evaluated using RMSE, MAE, and MAPE, together with R^2^ values (Figure S2 and Figure 12A). Based on the overall comparison, ExtraTrees showed the best performance for force prediction, whereas Bagging showed the highest predictive performance for distance prediction. The top-performing models were further compared by plotting predicted values against experimental measurements, and the predicted trends were generally consistent with the observed values (Figure 12B).

The selected models were further evaluated using goodness-of-fit and residual analyses. For force prediction, the ExtraTrees model showed good agreement between observed and predicted values, with an R^2^ of 0.86 (Figure 12C). The residuals were randomly distributed within the control limits, and the residual histogram showed an approximately normal-like distribution (Figure 12D,E). For distance prediction, the Bagging model also showed reliable predictive performance, with an observed–predicted R^2^ of 0.80 (Figure 12F). The residual plot and histogram showed no obvious systematic deviation, supporting the stability of the model for distance prediction (Figure 12G,H).

Feature-importance analysis further indicated that temperature, API content, and screw speed were the dominant variables affecting both force and distance, whereas HPC content and the number of mixing sections contributed less strongly (Figure 12I). The correlation heatmaps also showed that the effects of formulation and process variables differed between force and distance, highlighting the need for data-driven models to capture these nonlinear relationships (Figure 12J,K). The solid-state transition observed at high drug loading provides a possible mechanistic explanation for these nonlinear mechanical responses. At low drug loadings, LY-11h was molecularly dispersed within a predominantly single-phase amorphous polymer matrix, and filament mechanics were mainly governed by the continuous HPMC-AS/HPC-EF phase. As the drug loading approached 30%, residual crystalline or drug-rich domains introduced increasing structural heterogeneity. Such domains may alter stiffness and load transfer and, when interfacial adhesion with the polymer matrix is insufficient, may also act as local stress concentrators that promote crack initiation and reduce filament flexibility. The pronounced brittleness observed at 50% drug loading suggests that this defect-dominated behavior became increasingly important at high drug concentrations.

Accordingly, the influence of drug loading should not be interpreted solely as a continuous compositional effect, but as the combined consequence of drug concentration, amorphization efficiency, and phase heterogeneity. This transition may contribute to the threshold-like and non-monotonic relationships captured by the tree-based ensemble models. Because solid-state phase status was not included as an independent ML feature, the importance assigned to drug loading partly incorporates the mechanical consequences of residual crystallinity. Therefore, the current models are best regarded as empirical predictors within the investigated formulation–processing space, and future models may incorporate NIR-, DSC-, or PXRD-derived amorphous-fraction descriptors to improve mechanistic interpretability.

Based on the cross-validation results, ExtraTrees and Bagging were selected as the optimal models for predicting filament maximum force and distance at break, respectively. Because both models are tree-based ensemble methods, their predictions are generated by averaging the outputs of multiple base estimators rather than by a single closed-form regression equation. The general prediction form can be expressed as:

$$y\;=\frac{1}{M}\sum_{m=1}^{M}Tm(x)$$

where *y* is the predicted mechanical response, *M* is the number of base estimators, *Tm* is the prediction from the *m*-th estimator, and *x* represents the input feature vector composed of formulation and process variables. The trained ExtraTrees model was used for maximum force prediction, while the trained Bagging model was used for distance-at-break prediction. Feature-importance analysis was then applied to interpret the relative contribution of each variable to the predicted mechanical properties.

The superior performance of ExtraTrees for force prediction and Bagging for distance prediction suggests that filament mechanical behavior was governed by nonlinear interactions among drug loading, extrusion temperature, and screw speed. This observation is consistent with the RSM results, where the same variables showed interactive effects on maximum force and distance at break. Compared with the quadratic DoE model, the ensemble ML models were more flexible in describing nonlinear and non-monotonic relationships, particularly when the process variables simultaneously affected melt viscosity, drug–polymer dispersion, and filament structural integrity. Therefore, the ML analysis was not only used for numerical prediction but also provided an additional data-driven interpretation of the formulation-process-property relationship governing filament printability. The ML results provide a data-driven approach for predicting the printability-related mechanical properties of LY-11h ASD filaments, which may reduce trial-and-error formulation screening and accelerate the development of LY-11h dosage forms for precision dosing.

### 3.5. PAT Implementation

#### 3.5.1. NIR Method Development for Amorphous-Content Determination

**Figure 13:**
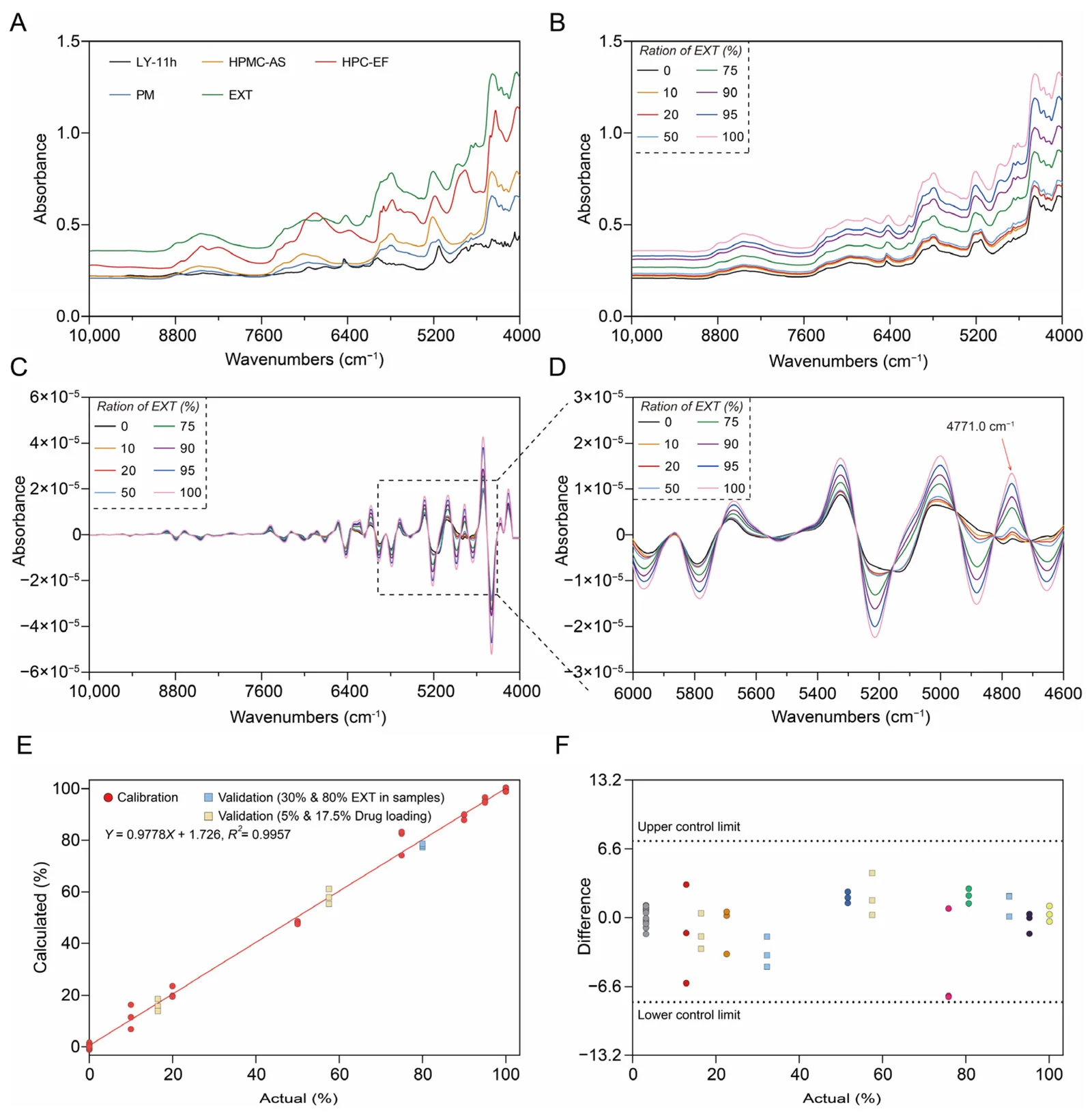
Development and validation of the NIR spectroscopy method for quantifying the amorphous fraction of LY-11h in ASD formulations. (**A**) Raw NIR spectra of individual components, PM, and EXT; (**B**) Raw NIR spectra of PM/EXT mixtures at different EXT proportions; (**C**) Second-derivative NIR spectra of PM/EXT mixtures; (**D**) Magnified view of the 4600–6000 cm^−1^ region; (**E**) Calibration curve of the NIR model; (**F**) Control chart for precision evaluation. In panel F, different colors represent samples with different actual EXT proportions, and different marker shapes distinguish the sample sets, with circles indicating calibration samples and squares indicating validation samples.

Offline NIR spectra of LY-11h, HPMC-AS, HPC-EF, PM, and EXT were collected to identify characteristic spectral features for amorphization quantification (Figure 13A,B). Because the spectra of LY-11h and the excipients showed substantial overlap, second-derivative treatment was applied to improve spectral resolution. Compared with PM, EXT exhibited clear spectral changes in the combination region of 4600–5200 cm^−1^, with a pronounced difference around 4771.0 cm^−1^ in the second-derivative spectra (Figure 13C,D). Therefore, the peak at 4771.0 cm^−1^ was selected as the characteristic signal for quantifying the amorphous fraction of LY-11h in the ASD formulations.

PM/EXT mixtures with different EXT proportions were then prepared to establish the calibration model. The second-derivative absorbance at 4771.0 cm^−1^ showed a clear correlation with EXT fraction, indicating good linearity for amorphous-content quantification (Figure 13E). This relationship was further verified using formulations with different LY-11h loadings of 5.0%, 17.5%, and 30.0%, which showed consistent spectral trends and proportional responses at the characteristic peak (Figure S3A,B). Validation samples containing 30% and 80% EXT also confirmed the applicable response range of the method (Figure S3C,D).

The precision and accuracy of the NIR method were further evaluated. Each sample was measured in triplicate, and the absorbance values at 4771.0 cm^−1^ were plotted in a control chart (Figure 13F). All data points fell within the upper and lower control limits, demonstrating good repeatability and precision. In addition, the predicted amorphous fractions of validation samples showed good agreement with the actual EXT proportions (Figure 13E), confirming the reliability of the established NIR model for quantifying LY-11h amorphization across different drug loadings and amorphization levels. The established NIR method enables quantitative assessment of LY-11h amorphization, which is essential for ensuring that the ASD formulation can maintain the amorphous drug state required to improve apparent solubility and oral dissolution performance.

#### 3.5.2. Real-Time Process Monitoring

**Figure 14:**
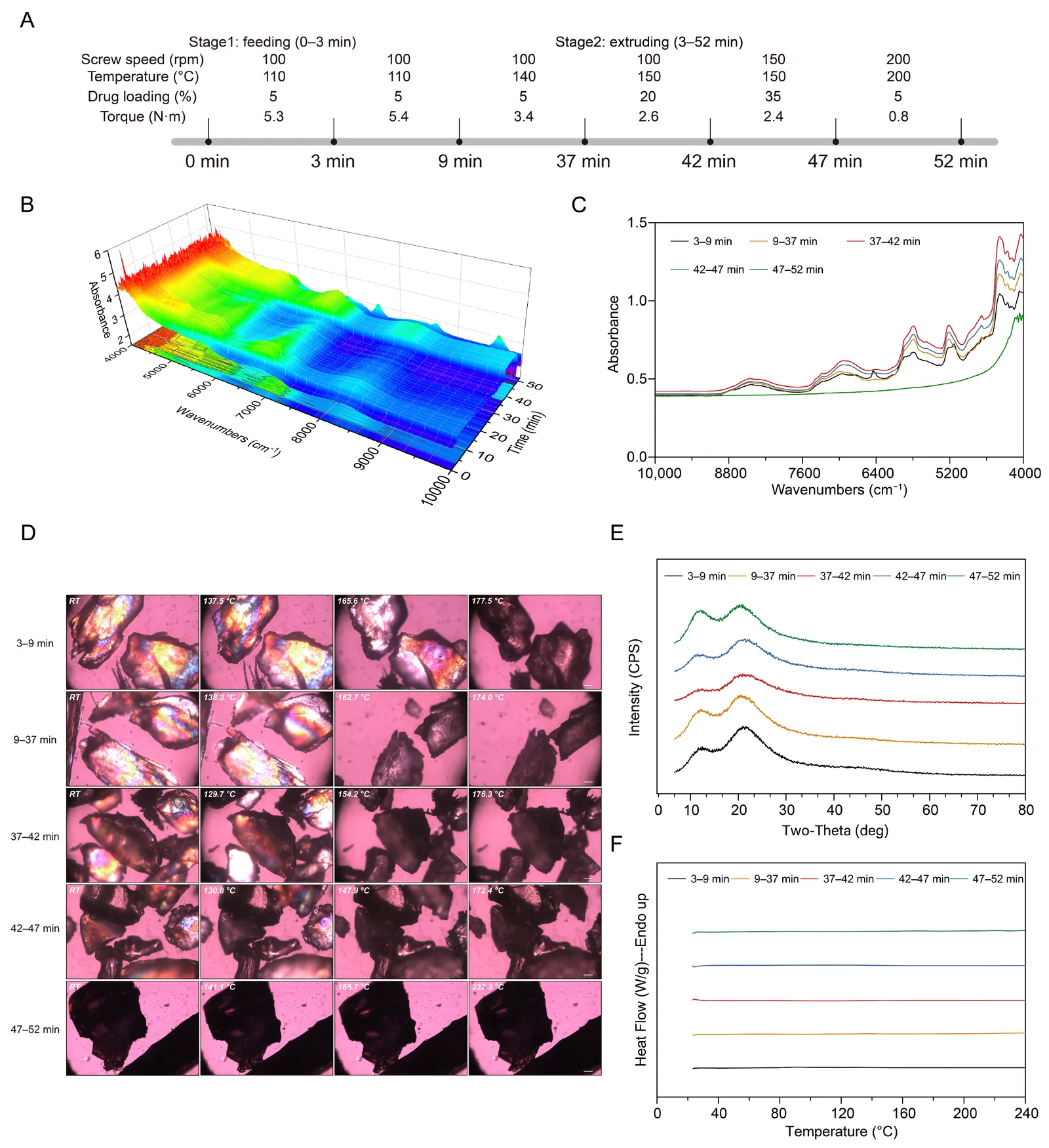
Real-time monitoring of the HME process. (**A**) Time-resolved changes in screw speed, barrel temperature, drug loading, and extrusion torque during the feeding and extrusion stages. The process progressed from initial feeding and material transition (0–9 min), through relatively stable extrusion (9–37 min), to sequential changes in temperature, drug loading, and screw speed (37–47 min), followed by a final high-temperature and high-shear stage (47–52 min). Torque decreased progressively as the process conditions changed. (**B**) 3D contour plot of real-time NIR spectra collected throughout the extrusion process. (**C**) Averaged NIR spectra of different time segments (3–9, 9–37, 37–42, 42–47, and 47–52 min). (**D**) PLM images of extrudates collected at different process stages. Scale bar = 100 μm. (**E**) PXRD patterns of extrudates collected during the five extrusion intervals. (**F**) DSC thermograms of the corresponding extrudates. In panel B, the color gradient represents the magnitude of NIR absorbance, with warmer colors indicating higher values and cooler colors indicating lower values. In panels C, E, and F, different colors distinguish the five extrusion time intervals.

An in-line PAT system based on NIR spectroscopy was implemented to monitor material transformation during the HME process. As shown in Figure 14A, the process was divided into an initial feeding stage and five subsequent extrusion intervals. During the initial feeding stage from 0 to 3 min, the formulation was introduced at 100 rpm, 110 °C, and 5% drug loading. The 3–9 min interval represented early extrusion and material transition under the same nominal conditions. From 9 to 37 min, relatively stable extrusion was achieved at 100 rpm, 140 °C, and 5% drug loading. The process conditions were then sequentially changed to 100 rpm, 150 °C, and 20% drug loading from 37 to 42 min and to 150 rpm, 150 °C, and 35% drug loading from 42 to 47 min. Finally, a high-temperature and high-shear condition of 200 rpm and 200 °C with 5% drug loading was applied from 47 to 52 min. The recorded torque progressively decreased from approximately 5.3–5.4 N·m during the initial stages to 0.8 N·m during the final stage, reflecting the combined effects of thermal softening, formulation composition, and shear conditions on melt-flow resistance. These deliberate stepwise changes in drug loading and processing conditions were introduced to evaluate the ability of the in-line NIR system to monitor formulation–process changes during continuous extrusion and to generate independent PAT-stage samples for validating the predictive performance of the DoE and ML models.

Real-time NIR spectra were continuously collected throughout the process (Figure 14B), and the averaged spectra from different time segments showed clear spectral variations, particularly in the 4000–6000 cm^−1^ region (Figure 14C). These changes were associated with variations in formulation composition, polymer melting, and the evolving molecular environment during extrusion, indicating that NIR spectroscopy could track process-dependent transitions in the HME system. PLM was further used to visualize the morphological and solid-state changes in extrudates collected at different process stages (Figure 14D). Samples from the early stage showed strong birefringence under polarized light, indicating pronounced optical anisotropy within the extruded polymer matrix. However, the PXRD patterns of extrudates collected during all five process intervals exhibited broad amorphous halos without distinct LY-11h diffraction peaks (Figure 14E), and the corresponding DSC thermograms showed no detectable melting endotherm attributable to crystalline LY-11h (Figure 14F). These complementary results indicated that no residual crystalline LY-11h was detectable within the sensitivity limits of PXRD and DSC, including in the sample collected during the earliest 3–9 min interval.

Therefore, the birefringence observed in the earlier-stage extrudates was not interpreted as direct evidence of residual drug crystallinity. Birefringence can also arise in amorphous polymeric materials from residual thermal or mechanical stresses and flow-induced polymer-chain orientation generated during extrusion and subsequent cooling [44,45]. As the processing conditions changed, the birefringence gradually weakened, suggesting a reduction in processing-induced optical anisotropy. During the final high-temperature stage, the extrudate became markedly darker and showed little visible birefringence. An effective NIR signal was also not obtained during part of this late processing period. The combination of sample darkening and loss of a reliable NIR response suggested possible thermal deterioration under the severe processing conditions. However, because dark or highly absorbing materials may also exhibit substantially reduced NIR reflectance, the absence of an effective NIR signal was not regarded as definitive evidence of chemical degradation.

Importantly, the intervals shown in Figure 10 represent sequential operating stages rather than the residence time of an individual material fraction inside the extruder. Therefore, the sample collected at 47–52 min had not remained in the extruder for approximately 52 min. The absence of detectable crystalline LY-11h in the 3–9 min sample demonstrated that completion of the full experimental sequence was not required to achieve amorphization.

#### 3.5.3. Validation of ML/DoE Predictions

Filament samples collected during PAT monitoring were used as an independent process-stage validation set to evaluate the prediction behavior of the DoE and ML models under dynamic HME conditions. Five representative extrusion stages were selected according to the time-resolved changes in extrusion temperature, screw speed, drug loading, and torque, as well as the corresponding material-state information obtained from in-line NIR spectroscopy and PLM observation. These PAT-stage samples covered different extrusion states, including early material transition, relatively stable extrusion, and high-temperature/high-shear processing stages. Therefore, they provided a useful dataset for assessing how the two modeling strategies performed when applied to process conditions with different degrees of similarity to the original BBD space.

The experimentally measured maximum force and distance at break were compared with the values predicted by the DoE and ML models, as summarized in Table 1. For samples collected within the original DoE design space, particularly the 9–37 min and 37–42 min stages, both models showed good agreement with the experimental measurements. This result confirms that the quadratic DoE response-surface model effectively described the relationship between formulation/process variables and filament mechanical properties within the experimental region defined by the BBD. The agreement between predicted and measured values also supports the reliability of the selected DoE factors, namely extrusion temperature, screw speed, and drug loading, as critical variables governing filament mechanical performance.

For the process stages outside the original DoE design space, the two modeling approaches showed different prediction behavior. The quadratic DoE model was developed to describe local factor–response relationships within the experimental region defined by the BBD. Consequently, its predictions became increasingly sensitive to the squared and interaction terms when applied to factor combinations outside that region. At the 47–52 min stage, this polynomial behavior resulted in a negative predicted force, which is physically inadmissible. This value should therefore be interpreted as an indication that the model had been applied beyond its reliable domain rather than as a realizable mechanical response.

For practical implementation, model-domain assessment should precede interpretation of the predicted response. The input formulation and processing conditions should first be compared with the factor ranges and boundaries used for model development. Predictions generated outside the calibrated region should be flagged as extrapolative and subjected to experimental confirmation or model updating. Physically impossible outputs could additionally be prevented through constrained regression or a positive-response transformation during model construction. However, such output constraints would function only as secondary safeguards and would not restore predictive reliability when the input conditions are substantially outside the original design space.

In contrast, the tree-based ensemble ML models produced physically plausible predictions that remained close to the measured values across the tested PAT stages. Unlike an unrestricted quadratic surface, tree-based models assign new input conditions to terminal regions defined by the training data, and their predictions are derived from the observed responses represented within those regions. This bounded prediction behavior reduced the risk of rapid numerical divergence under the present dynamic conditions. In addition, the ML models were trained using the broader combined dataset from the single-factor and BBD studies. Their improved performance therefore reflects both their flexibility in capturing nonlinear relationships and the broader representation of the investigated formulation–processing space. Nevertheless, tree-based models do not provide unrestricted physical extrapolation, and their apparently plausible outputs should not be assumed to remain reliable under conditions substantially different from those represented in the training dataset. Thus, the present results demonstrate greater predictive robustness of the selected ML models for this PAT-stage validation rather than universal superiority of ML over DoE.

The PAT observations provided additional material-state information for interpreting the prediction results. During the early extrusion stage, stronger birefringence was observed by PLM, which was consistent with incomplete melt homogenization and/or stress- or flow-induced molecular orientation relative to the stable extrusion stage. Because no annealing experiment was performed, these possible contributions could not be distinguished conclusively. During the later high-temperature/high-shear stage, reduced birefringence together with a darker extrudate appearance indicated that the material had entered a state different from that observed during stable extrusion and suggested possible changes associated with the more severe thermal–mechanical history. These observations help explain why PAT-stage samples could exhibit different mechanical behavior even when some nominal process variables were similar to those represented in the model-development datasets. Thus, PAT contributed material-state context that could not be captured by the nominal formulation and processing variables alone.

Prediction performance was further evaluated using R^2^, MAE, and RMSE values calculated across all five PAT validation points. The ML models showed strong overall agreement with the experimental measurements, with R^2^ values of 0.99 for maximum force and 0.96 for distance at break. The corresponding MAE values were 75.32 gf and 0.064 mm, and the RMSE values were 80.33 gf and 0.065 mm, respectively. In contrast, the aggregate DoE validation metrics were strongly affected by the three PAT stages located outside the original BBD region, particularly the physically inadmissible force prediction at 47–52 min. Although pointwise agreement remained reasonable for the two stages located within the design space, the poor aggregate performance across all five samples demonstrates that the quadratic model should not be used operationally without first assessing whether the input conditions fall within its calibrated domain. These results highlight the complementary roles of the two approaches: DoE provides interpretable main and interaction effects within a predefined formulation–processing region, whereas ML offers greater predictive flexibility across the broader conditions represented in its training dataset.

Overall, the PAT-stage validation demonstrated the complementary roles of DoE, ML, and PAT in formulation–process–property analysis. DoE established interpretable factor–response relationships within the designed experimental space, ML improved prediction of filament mechanical performance across the tested dynamic process conditions, and PAT supplied real-time material-state information to support interpretation of model behavior. Incorporating explicit model-domain and physical-plausibility checks into this integrated workflow would further ensure that predictions requiring experimental confirmation are identified before they are used for process decisions. This strategy provides a rational basis for selecting suitable HME conditions and improving the reliability of filament-performance prediction for downstream FDM 3D printing.

### 3.6. Filament Processability and pH-Dependent Drug Release from 3D Printed Tablets

**Figure 15:**
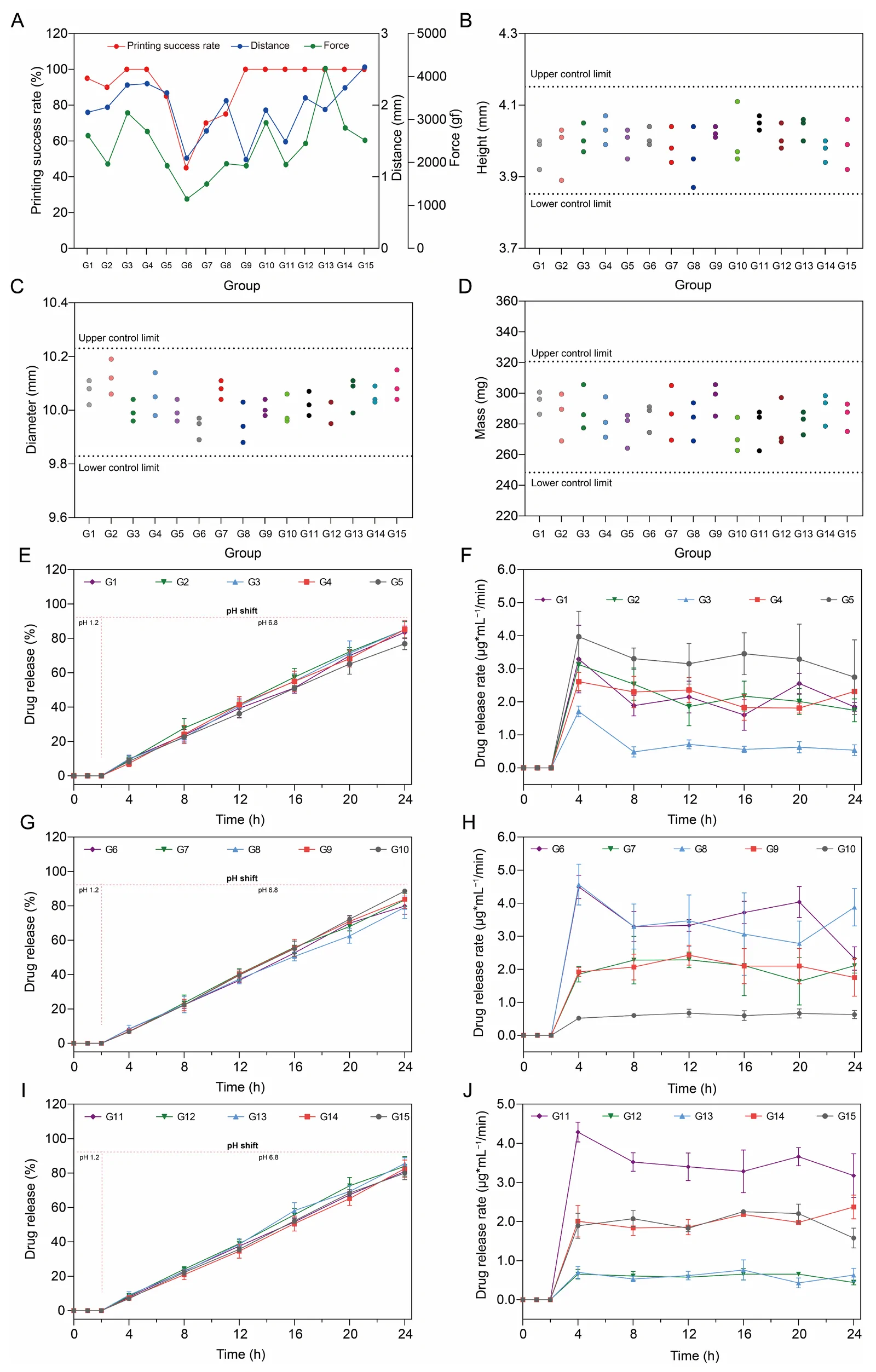
3D printing performance and in vitro release of 15 groups of DoE-designed filaments. (**A**) Printing success rate, filament distance, and force across all groups. (**B**–**D**) Control charts for printed tablet height, diameter, and mass, respectively. (**E**,**G**,**I**) In vitro drug release profiles of G1–G5, G6–G10, and G11–G15, respectively. (**F**,**H**,**J**) In vitro drug release rates of G1–G5, G6–G10, and G11–G15, respectively. In panels (**B**–**D**), the different colors of the circles represent different filament groups (G1–G15). In panels E, G, and I, the red dashed line indicates the pH-shift point, at which the dissolution medium was changed from pH 1.2 to pH 6.8.

The printability of the 15 BBD-designed HME filaments was systematically evaluated, together with the quality consistency and in vitro release behavior of the printed dosage forms. All 15 BBD formulations (G1–G15) were fabricated into printed tablets and included in the in vitro release study. Dissolution testing was therefore not restricted to a single selected formulation. No single final optimized formulation was defined; instead, the purpose was to compare printability and pH-responsive release behavior across the BBD space and to identify a practical formulation–processing region with balanced overall performance. As shown in Figure 15A, the printing success rate varied markedly among the formulations, ranging from approximately 45% to 100%. Formulations with higher distance at break and appropriate maximum force generally showed better printability, whereas mechanically weak or brittle filaments were more prone to feeding instability, nozzle clogging, or filament breakage. Among the tested groups, G6 showed the lowest printing success rate, while several formulations with more balanced mechanical properties achieved printing success rates of 100%.

The dimensional and mass profiles of the printed tablets reflected formulation-dependent differences in filament feeding and deposition behavior during FDM printing (Figure 15B–D). All formulations remained within the upper and lower control limits, indicating good process repeatability and acceptable quality uniformity. Tablet diameter and height showed relatively narrow distributions across the tested formulations, indicating stable geometric reproduction under the selected printing parameters. In contrast, tablet mass showed a broader distribution in some groups, which can be attributed to the combined influence of filament mechanical performance, melt flow behavior, and material delivery during extrusion-based printing [46,47]. Formulations with balanced maximum force and distance at break generally produced more stable printing outcomes, supporting the relevance of filament mechanical properties as critical predictors of FDM processability. The printed tablets also showed relatively smooth and homogeneous surfaces (Figure S4).

In vitro release testing demonstrated pH-dependent biphasic release behavior across the formulations, with minimal release in acidic medium followed by gradual release after the pH shift to 6.8 (Figure 15E–J). A direct pH-shift dissolution comparison further showed that, unlike pure LY-11h and the corresponding physical mixture, the hot-melt extrudate markedly restricted drug release during the initial acidic stage and achieved rapid, near-complete release after transition to pH 6.8, supporting the pH-responsive release function of the HPMC-AS-based ASD system (Figure S5). The corresponding release-rate profiles showed that drug release increased rapidly after the pH shift and then proceeded at broadly comparable rates, with only modest numerical variation among the formulations. Despite these formulation-dependent differences in mass distribution, all printed tablets maintained the intended cylindrical geometry and showed reproducible pH-responsive release behavior. The limited release in acidic medium followed by accelerated release at pH 6.8 was consistent with the pH-dependent solubility of HPMC-AS and confirmed that the HME-FDM process preserved the functional release characteristics of the ASD system. Although minor numerical differences were observed in the calculated release-rate profiles, the overall release trends were broadly comparable, and most formulations achieved substantial drug release within 24 h. These results confirm that HME-FDM processing can convert LY-11h ASD filaments into reproducible 3D-printed tablets with pH-responsive release behavior, supporting a formulation strategy that may improve intestinal delivery while enabling flexible dose design for a narrow-therapeutic-window AML drug candidate.

### 3.7. Drug-Release Kinetics and Mechanism

The drug-release profiles of the 15 DoE formulations were fitted using zero-order, first-order, Higuchi, Korsmeyer–Peppas, and Peppas–Sahlin models, and the corresponding kinetic parameters are summarized in Table 2. The zero-order model showed consistently high coefficients of determination across all formulations, with R^2^ values ranging from 0.9985 to 0.9999. These results indicated that LY-11h was released from the 3D-printed tablets at an approximately constant rate over the investigated release period. The fitted zero-order release constants (K_0_) ranged from 3.577 to 3.998 across the 15 formulations, corresponding to only an approximately 1.12-fold range. Thus, the kinetic analysis indicated modest numerical variation in the apparent release rate rather than clearly distinct release-rate behavior among the formulations.

The Korsmeyer–Peppas model also provided excellent fitting, with R^2^ values ranging from 0.9976 to 0.9999. The release exponent (n) ranged from 0.892 to 1.068. For cylindrical dosage forms, n values close to 0.89 indicate Case II transport, whereas values greater than 0.89 are generally associated with super Case II transport. Therefore, the release of LY-11h from the printed tablets predominantly followed Case II or super Case II behavior, suggesting that polymer-chain relaxation and matrix erosion were the principal processes governing drug release rather than simple Fickian diffusion alone.

Although the LY-11h loading varied from 5.0% to 30.0% among the DoE formulations, consistently high zero-order fitting coefficients and comparable Korsmeyer–Peppas release exponents were observed. No systematic change in the release model or transport mechanism was identified with increasing drug loading. Thus, within the investigated formulation range, drug loading did not significantly alter the predominant zero-order, Case II-controlled release behavior of the printed tablets. Collectively, these findings demonstrate that the 3D-printed tablets maintained a robust and predictable drug-release mechanism across the investigated drug-loading range, supporting the reproducibility of the developed formulation and printing process.

## 4. Conclusions

In this study, an integrated formulation and manufacturing strategy for precisely controlled LY-11h drug delivery system development was established by combining HME and FDM 3D printing technologies. Single-factor screening showed that HPMC-AS grade, HPC-EF content, and screw configuration influenced filament solid-state characteristics, mechanical performance, and processability, whereas the DoE analysis established statistically significant response models describing the combined and interactive effects of drug loading, extrusion temperature, and screw speed on key filament properties. Through systematic formulation and process optimization, printable LY-11h ASD filaments with suitable physicochemical properties, mechanical robustness, and FDM printability were successfully prepared. In addition, in-line NIR spectroscopy combined with PLM established a PAT strategy for real-time monitoring of LY-11h amorphization and melt homogenization during HME, while ML models improved the prediction of key filament mechanical properties. These tools enhanced process understanding and manufacturing control, supporting the reliable preparation of LY-11h ASD filaments for downstream 3D printing.

The developed HME–FDM platform enabled the reproducible fabrication of LY-11h dosage forms with controllable geometric attributes and pH-responsive release behavior. These findings establish a technological proof of concept for variable-dose manufacturing rather than direct evidence of individualized pharmacokinetic control. Specifically, the HPMC-AS-based ASD strategy substantially improved the intestinal-pH performance of LY-11h. In the equilibrium-solubility study, the solubility of LY-11h at pH 6.8 increased from 2.8 ± 1.1 μg/mL for the pure drug to 224.1–239.1 μg/mL for the HPMC-AS-based formulations, corresponding to an approximately 80.0- to 85.4-fold enhancement. In the direct pH-shift dissolution comparison, the hot-melt extrudate restricted LY-11h release during the initial acidic stage and subsequently achieved progressive, near-complete release after transition to pH 6.8, whereas pure LY-11h and the physical mixture showed markedly lower apparent release after the pH shift. Meanwhile, FDM 3D printing provided a flexible manufacturing approach for modulating dose and release behavior, thereby supporting the development of individualized dosage forms for a narrow-therapeutic-window AML drug candidate. Together, the equilibrium-solubility and dissolution results demonstrate that the ASD-based HME–FDM strategy enhanced LY-11h solubility at intestinal pH and enabled reproducible pH-responsive release from the printed dosage forms.

Future studies will therefore develop defined LY-11h dose-strength series and evaluate drug-content uniformity, biorelevant dissolution, in vivo pharmacokinetics, and dose–exposure relationships. Pharmacodynamic efficacy, safety, and interindividual variability will also need to be investigated to determine whether changes in tablet geometry or composition can produce predictable systemic exposure within the desired therapeutic range. These studies will be essential before the present manufacturing platform can be considered suitable for individualized dose selection or precision pharmacotherapy.

## Figures and Tables

**Table 1:** Validation of DoE and ML predictions using PAT-stage filament samples.

Stage	Actual		DoE Predicted Value		ML Predicted Value	
	Force (gf)	Distance (mm)	Force (gf)	Distance (mm)	Force (gf)	Distance (mm)
3–9 min	1340.6	1.34	2694.2	1.18	1452.9	1.38
9–37 min	3978.1	2.07	4140.9	2.01	4007.5	2.14
37–42 min	2782.3	1.95	2690.8	1.88	2865.4	2.02
42–47 min	1682.4	1.25	2018.0	2.05	1619.9	1.18
47–52 min	1523.1	1.62	−1827.7	1.40	1612.4	1.55

Note: The 3–9 min, 42–47 min, and 47–52 min stages were outside the original DoE design space, whereas the 9–37 min and 37–42 min stages were within the DoE design space. Prediction performance was evaluated using all five PAT validation points. For maximum force, the DoE model showed R^2^ = −1.67, MAE = 1058.86 gf, and RMSE = 1625.28 gf, whereas the ML model showed R^2^ = 0.99, MAE = 75.32 gf, and RMSE = 80.33 gf. For distance at break, the DoE model showed R^2^ = −0.38, MAE = 0.262 mm, and RMSE = 0.380 mm, whereas the ML model showed R^2^ = 0.96, MAE = 0.064 mm, and RMSE = 0.065 mm. The negative DoE-predicted force is retained as the raw unconstrained polynomial output for transparency. It is physically inadmissible and indicates that the corresponding process condition was outside the applicable domain of the BBD response-surface model.

**Table 2:** Kinetic parameters and coefficients of determination for different drug-release models fitted to the DoE formulations.

#	Zero-Order		First-Order		Higuchi		Korsmeyer-Peppas			Peppas-Sahlin			
	K_0_	R^2^	k	R^2^	k	R^2^	k_kp_	n	R^2^	k_1_	k_2_	m	R^2^
R1	3.817	0.9990	0.059	0.9838	14.943	0.8920	4.185	0.968	0.9980	5.264	0.045	0.82	0.9985
R2	3.998	0.9985	0.065	0.9899	15.723	0.8715	5.422	0.892	0.9994	5.458	<0.001	0.85	0.9994
R3	3.922	0.9994	0.062	0.9844	15.342	0.8621	4.258	0.971	0.9988	4.779	0.019	0.89	0.9965
R4	3.885	0.9991	0.061	0.9874	15.207	0.8949	4.384	0.957	0.9987	4.800	0.014	0.89	0.9946
R5	3.577	0.9995	0.054	0.9905	14.021	0.9017	4.239	0.940	0.9996	4.606	0.012	0.89	0.9994
R6	3.732	0.9988	0.057	0.9864	14.590	0.8884	3.974	0.978	0.9977	4.469	0.016	0.89	0.9959
R7	3.845	0.9990	0.060	0.9890	15.048	0.8945	4.362	0.956	0.9987	4.712	0.008	0.89	0.9945
R8	3.583	0.9993	0.054	0.9886	14.037	0.8984	4.108	0.952	0.9994	4.747	0.027	0.86	0.9991
R9	3.889	0.9993	0.061	0.9868	15.189	0.8864	4.084	0.983	0.9987	4.617	0.018	0.89	0.9923
R10	3.989	0.9998	0.063	0.9801	15.519	0.8695	3.479	1.048	0.9998	4.564	0.013	0.89	0.9907
R11	3.708	0.9999	0.057	0.9873	14.501	0.8911	3.959	0.977	0.9999	4.485	0.021	0.89	0.9981
R12	3.923	0.9992	0.062	0.9860	15.347	0.8927	4.331	0.965	0.9986	4.777	0.013	0.89	0.9903
R13	3.919	0.9988	0.062	0.9840	15.321	0.9608	4.164	0.979	0.9976	4.702	0.019	0.89	0.9921
R14	3.656	0.9992	0.055	0.9804	14.218	0.8657	3.010	1.068	0.9994	3.860	0.044	0.89	0.9966
R15	3.693	0.9993	0.056	0.9862	14.411	0.8811	3.650	1.004	0.9987	4.249	0.022	0.89	0.9915

## Data Availability

The original contributions presented in this study are included in the article/Supplementary Materials. Further inquiries can be directed to the corresponding authors.

## Supplementary Materials

The following supporting information can be downloaded at: https://www.mdpi.com/article/10.3390/pharmaceutics18081002/s1, Figure S1: Hot-stage polarized light microscopy (PLM) images of physical mixtures and DoE-designed extrudates with different drug loadings; Figure S2: Performance evaluation of machine learning algorithms for predicting force and distance; Figure S3: NIR spectra of LY-11h extrudates at different drug loadings; Figure S4: Micrographs of 3D printed tablets produced from DoE filaments; Figure S5: pH-shift dissolution profiles of pure LY-11h, the physical mixture, and the corresponding hot-melt extrudate; Table S1: Box-Behnken design of 15 groups; Table S2: The fitting models of the correlation between individual factors (temperature, screw speed, and drug loading) and response; Table S3: The mathematical models of correlation between the individual factors and distance; Table S4: The mathematical models of correlation between the individual factors and force; Table S5: Equilibrium solubility of pure LY-11h and extruded formulations prepared under different single-factor conditions [48,49,50,51,52].
