# Supplementary material for: Precision Drug Delivery of LY-11h for Acute Myeloid Leukemia Treatment Using Machine Learning-Assisted Hot Melt Extrusion and 3D-Printed Technologies
Source: Pharmaceutics. 2026 Aug 13;18(8):1002. doi: 10.3390/pharmaceutics18081002 (PMC13516310; doi:10.3390/pharmaceutics18081002)
Supplement: Supplementary file 1 [file pharmaceutics-18-01002-s001.zip › pharmaceutics-4451967-supplementary.pdf]

Supplement information

Methods

Thermalgravimetric analysis

Thermogravimetric analysis (TGA) was employed to investigate the thermal degradation behavior of both the pure drug LY-11h and its physical mixtures (PM). Samples (2–5 mg) were meticulously weighed and placed in aluminum pans prior to heating from 30 °C to 400 °C at a constant ramp of 20 °C per minute. All experiments were conducted under an inert atmosphere, which was maintained by a continuous flow of ultra-pure nitrogen. The resulting data were collected and analyzed using Microsoft Excel (version 2310, build 16.0.16924.20054).

Rheological characterization

Rheological characterization was determined using a Thermo HAAKE MARS 60 rotational rheometer (Thermal Fisher Scientific, Shanghai, China) equipped with an 8 mm parallel-plate geometry (P8 rotor and 8 mm lower plate). Samples (approximately 0.5 g) were loaded between the pre-heated plates and equilibrated at 100 °C for 5 min to ensure uniform preheating and contact. Dynamic temperature sweeps were conducted from 100 to 200 °C at a heating rate of 2 °C·min-1, using a constant strain of 0.1% and a fixed frequency of 1 Hz. The storage modulus (G'), loss modulus (G"), complex viscosity (η*) and loss tangent (tan δ = G"/G') were recorded continuously.

Differential scanning calorimetry (DSC)

Solid-state characterization of the raw materials, PM, hot-pressed samples, and milled extruded filaments (EXT) was performed using a DSC 200 F3 instrument (NETZSCH Gerätebau GmbH, Bavaria, Germany). Samples weighing between 5 and 15 mg were meticulously weighed into standard aluminum pans and hermetically sealed with aluminum lids (DSC consumables, Minnesota, USA). With the exception of the PM, all samples were analyzed within a temperature range of 40 °C to 200 °C at a heating rate of 20 °C/min. All data obtained were processed and graphically presented using Microsoft Excel (Version 2310

Build 16.0.16924.20054).

Powder X-ray Diffraction (PXRD)

The crystallographic properties of the raw materials, PM, the hot-pressed samples, and EXT were investigated by PXRD. The analysis was conducted using a D/max-2200PC benchtop diffractometer (Rigaku Corporation, Tokyo, Japan). Samples were mounted on standard sample holders and scanned over a 2θ range of 5° to 50°, with a scanning speed of 2°/min, a step size of 0.02°, and a resolution of 0.0025°. The instrument was operated at 45 kV and 15 mA during all measurements. All diffraction data were collected and processed using Microsoft Excel (Version 2310, Build 16.0.16924.20054).

Hot-Stage Polarized Light Microscopy (PLM)

A CX40P polarized light microscope (Ningbo ShunYu Analytical Instrument Co., Ltd., Yuyao, China) equipped with a hot stage (Linkam Scientific Instruments Ltd., Salfords, UK) was employed to observe the melting behaviors of raw materials, PM, the hot-pressed samples, and EXT. The samples were meticulously distributed on glass slides, with any surplus powder being meticulously removed. Subsequently, a coverslip was placed over the samples, which were then mounted on the microscope stage and observed under a 10× magnification. The analysis process entailed subjecting the samples to a heating protocol, whereby they were elevated from their initial room temperature to their respective melting points at a constant rate of 20 °C/min. The images were captured using a dedicated digital camera (EP-SUF880, Markham, Canada) combined with a 530 nm compensator (U-TP530, Olympus Corporation, Tokyo, Japan) under both bright and dark field conditions.

Fourier transform infrared spectroscopy (FTIR)

A comprehensive analysis was conducted using near-infrared spectroscopy (NIR) to examine raw materials, physical mixtures (PM), and extruded samples (EXT). This analysis was performed using an Antaris II NIR spectrometer (Thermo Scientific, UK). All samples were measured with consistent spectral collection parameters. The scanning wavenumber range was set at 4000–10000 cm^{-1}, with a spectral resolution of 8 cm^{-1}. The final spectrum for each iteration was obtained by averaging 32 individual scans, with the objective of reducing

baseline noise. Spectral data were collected and processed using Omnic 9 software (Thermo Scientific, UK) for subsequent qualitative and quantitative analysis.

Three-point bend

The flexibility and brittleness of the extruded filaments were assessed using a TA-XT2 texture analyzer (Texture Technologies Corporation, New York, NY) equipped with a TA-92N miniature three-point bending fixture with a 15 mm support span. The filaments were then divided into 20 mm segments and positioned centrally on the designated test fixture. The velocities of the pre-test, test, and post-test were set at 10 mm/s, 1 mm/s, and 10 mm/s, respectively. The probe was operated in distance-controlled mode, applying force to the filament over a 15 mm displacement distance. The collection and analysis of breaking distance, load force and stress data was conducted using Exponent software (Stable Micro Systems, Godalming, Surrey, UK).

Equilibrium solubility measurement

The equilibrium solubility of LY-11h and the corresponding formulations was determined in simulated gastric fluid (SGF, pH 1.2) and simulated intestinal fluid (SIF, pH 6.8) at 37 ± 0.5 °C. The media were prepared with reference to the USP–NF specifications for Simulated Gastric Fluid TS and Simulated Intestinal Fluid TS [48,49]. The experimental procedure was designed with reference to current recommendations for the characterization of apparent solubility and dissolution behavior of amorphous solid dispersions [8]. An excess amount of each sample was added to 20 mL of the corresponding medium and stirred at 200 rpm for 24 h. The suspensions were subsequently allowed to stand for a further 24 h to facilitate sedimentation of undissolved material. The supernatant was collected, appropriately diluted, and quantified by HPLC. All measurements were performed in triplicate (n = 3). Statistical analysis was performed using GraphPad Prism version 9.4 (GraphPad Software, San Diego, CA, USA), with one-way ANOVA followed by Tukey's multiple-comparison test.

In vitro drug release study

In vitro drug release was evaluated in triplicate using a Chinese Pharmacopoeia Apparatus II paddle system (RC8MD, TIANDA TIANFA, Tianjin, China). The apparatus

configuration and operating principles were based on the pharmacopoeial paddle method, whereas the two-stage pH-shift protocol was developed specifically for the present HPMC-AS-based ASD formulations with reference to USP General Chapters <711> and <1092> and current recommendations for dissolution-method development for amorphous solid dispersions [8,50-52].

Each vessel contained 500 mL of prewarmed simulated gastric medium consisting of 0.1 M HCl at pH 1.2 without pepsin, maintained at 37 ± 0.5 °C. The paddle speed was set at 100 rpm. One tablet was placed in each vessel, and samples were collected at 5, 15, 30, 60, and 120 min. After 2 h, the medium was adjusted to pH 6.8 by the controlled addition of sodium hydroxide and potassium dihydrogen phosphate while maintaining the temperature at 37 ± 0.5 °C. Paddle rotation was then resumed, and additional samples were collected at 240, 480, 720, 960, 1200, and 1440 min. LY-11h concentrations were quantified by HPLC. All experiments were performed in triplicate, and the results are presented as mean ± SD.

Release kinetic studies of printed tablets

The dissolution kinetics were modelled using the zero-order model, first-order model, Higuchi model, Korsmeyer-Peppas model, and Peppas-Sahlin model (Table). The accuracy of each model was assessed using the correlation coefficient (R^2).

Table. The formula and definition of different mathematical models.

Mathematical models	***Formula***	***Definition***
**Zero-order**	$Q_t = Q_0 + K_0 * t$	***Q_t***: the amount of drug released in time ***t***<br>***Q_0***: the initial amount of drug in the solution<br>***K_0*****:** the zero-order release constant
First-order	$log\, C = log\, C_0 - kt/2.303$	***C***: the amount of drug at time ***t***<br>***C_0***: the initial concentration<br>***k***: the first order rate constant
Higuchi	$\frac{Q_t}{Q_\infty} = k * t^{1/2}$	***Q_t***: the drug released at time ***t***

		Q_∞: the drug loading of the dosage<br>K: Higuchi constant
Korsmeyer-Peppas	$\frac{Q_t}{Q_\infty} = k * t^n$	Q_t: the drug released at time t<br>Q_∞: the drug loading of the dosage<br>k: the rate constant<br>n: the release exponent
Peppas-Sahlin	$\frac{Q_t}{Q_\infty} = k_1 * t^m + k_2 * t^{2m}$	Q_t: drug released at time t<br>Q_∞: is the drug loading of the dosage<br>k_1/k_2: the kinetic constant<br>m: the release exponent

High performance liquid chromatography (HPLC)

LY-11h was quantified using an Agilent 1260 Infinity II HPLC system equipped with a UV detector (Agilent Technologies, Santa Clara, CA, USA). Chromatographic separation was performed using a Diamonsil Plus C18 column (5 µm, 250 × 4.6 mm; DiKMA, Beijing, China). The chromatographic procedure and system-suitability assessment were developed with reference to the general principles described in USP General Chapter <621> and ICH Q14. Analytical-performance characteristics relevant to quantitative sample analysis were evaluated with reference to ICH Q2(R2)].

The injection volume was 5 µL. The mobile phase consisted of solvent A, an aqueous solution containing 8 mM KH_2PO_4 and 2 mM K_2HPO_4, and solvent B, acetonitrile. Gradient elution was performed as follows: 10–80% B from 0 to 35 min, 80–10% B from 35 to 36 min, followed by equilibration at 10% B from 36 to 45 min. The flow rate was 1.0 mL/min, the column temperature was maintained at 25 °C, and the autosampler temperature was maintained at 5 °C. LY-11h was detected at 220 nm. Data acquisition and processing were performed using Agilent software version C.01.07 SR2.

PAT tools implementation

Specificity: The LY-11h, HPMC-AS, HPC-EF, physical mixture (PM), and EXT prepared by HME were subjected to off-line NIR detection.

linearity and range: EXT (30% drug loading) was mixed with PM in the ratios of 0%, 10%, 20%, 50%, 75%, 90%, 95% and 100%. Subsequently, NIR was employed for detection purposes to determine the ASD content, and a linear regression equation was established.

Precision and accuracy: Samples were analyzed with three replicate measurements by off-line NIR spectroscopy and the associated errors were calculated. The ASD content was then computed in accordance with the established formula. 30% and 80% w/w of ASD content were precisely weighted and mixed with 80% of PM, and then the offline NIR spectrum was collected to validate the accuracy of the NIR measurement.

Figures

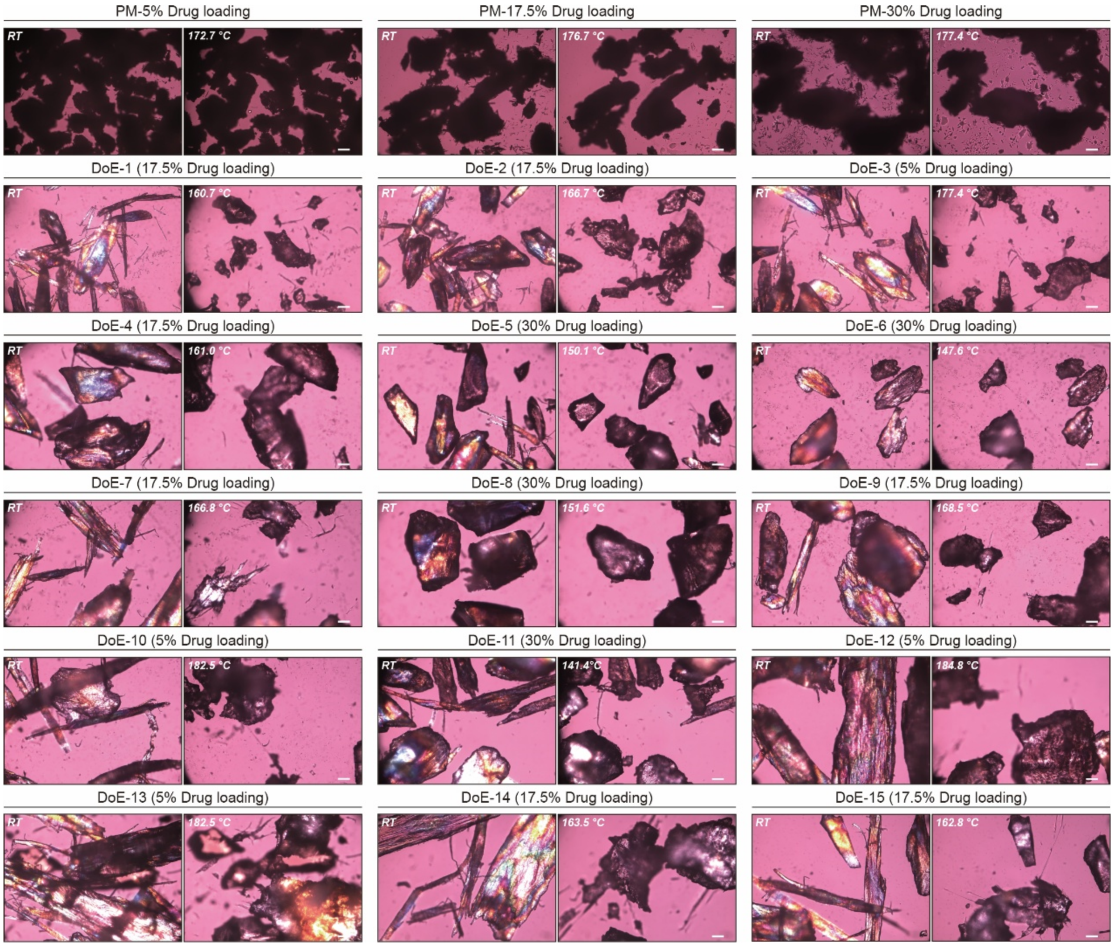

Figure S1. Hot-stage polarized light microscopy (PLM) images of physical mixtures and DoE-designed extrudates with different drug loadings. Scale bar represents 100 μm.

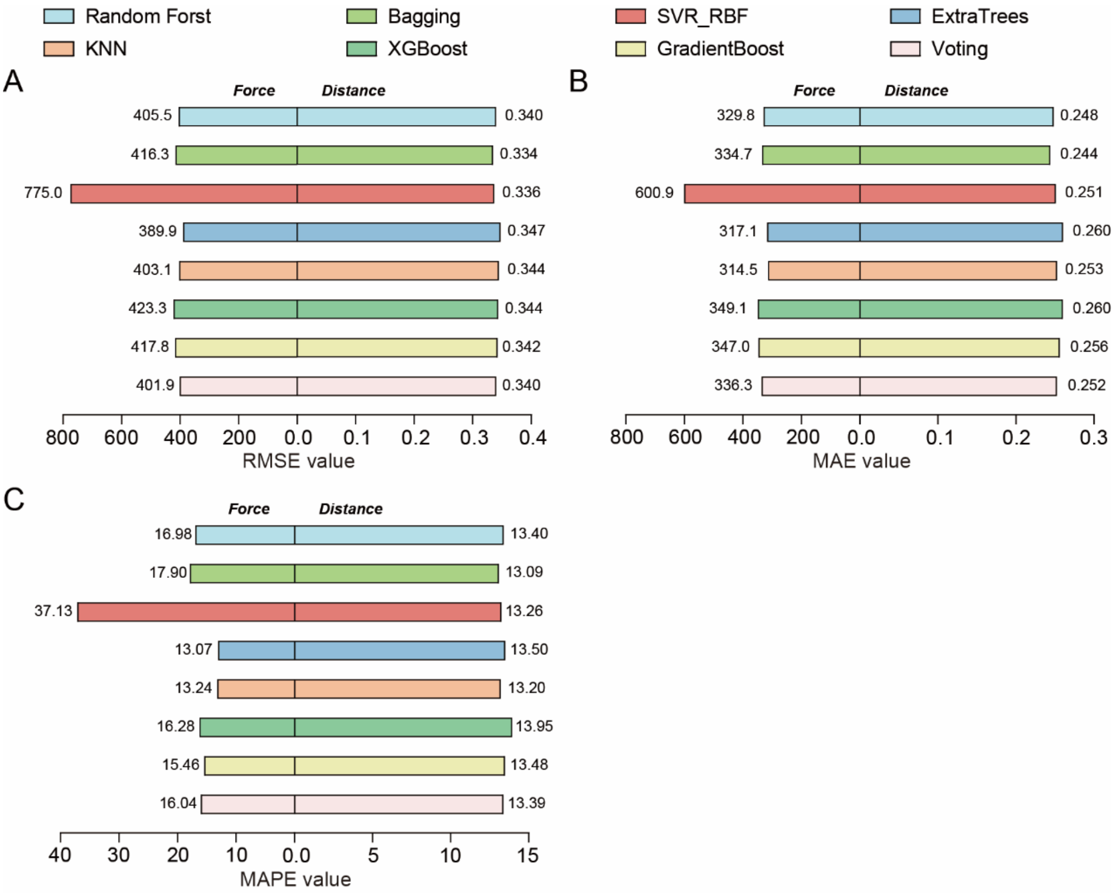

Figure S2. Performance evaluation of machine learning algorithms for predicting force and distance. (A) RMSE values of different models for force and distance prediction. (B) MAE values of different models for force and distance prediction. (C) MAPE values of different models for force and distance prediction.

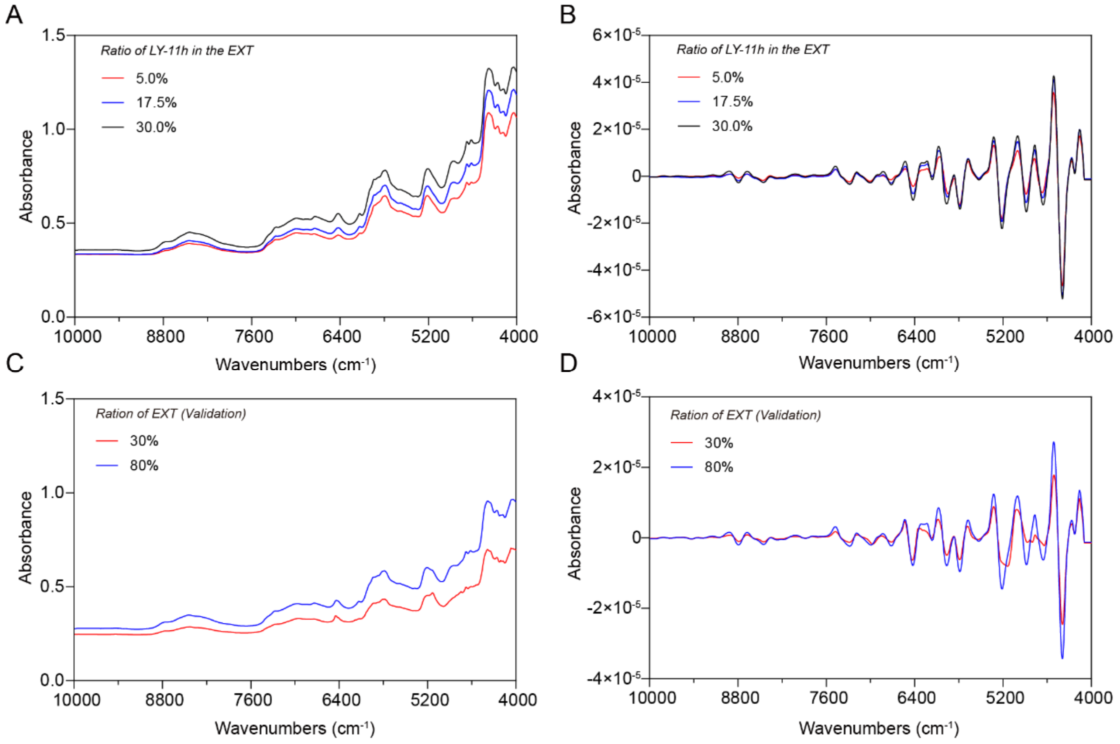

Figure S3. NIR spectra of LY-11h extrudates at different drug loadings. (A) Raw near-infrared (NIR) absorption spectra of calibration extrudates with LY-11h loadings of 5.0%, 17.5%, and 30.0%; (B) Second-derivative preprocessed NIR spectra of the calibration set; (C) Raw NIR spectra of validation set extrudates with different EXT ratios (30% and 80%); (D) Second-derivative preprocessed NIR spectra of the validation set.

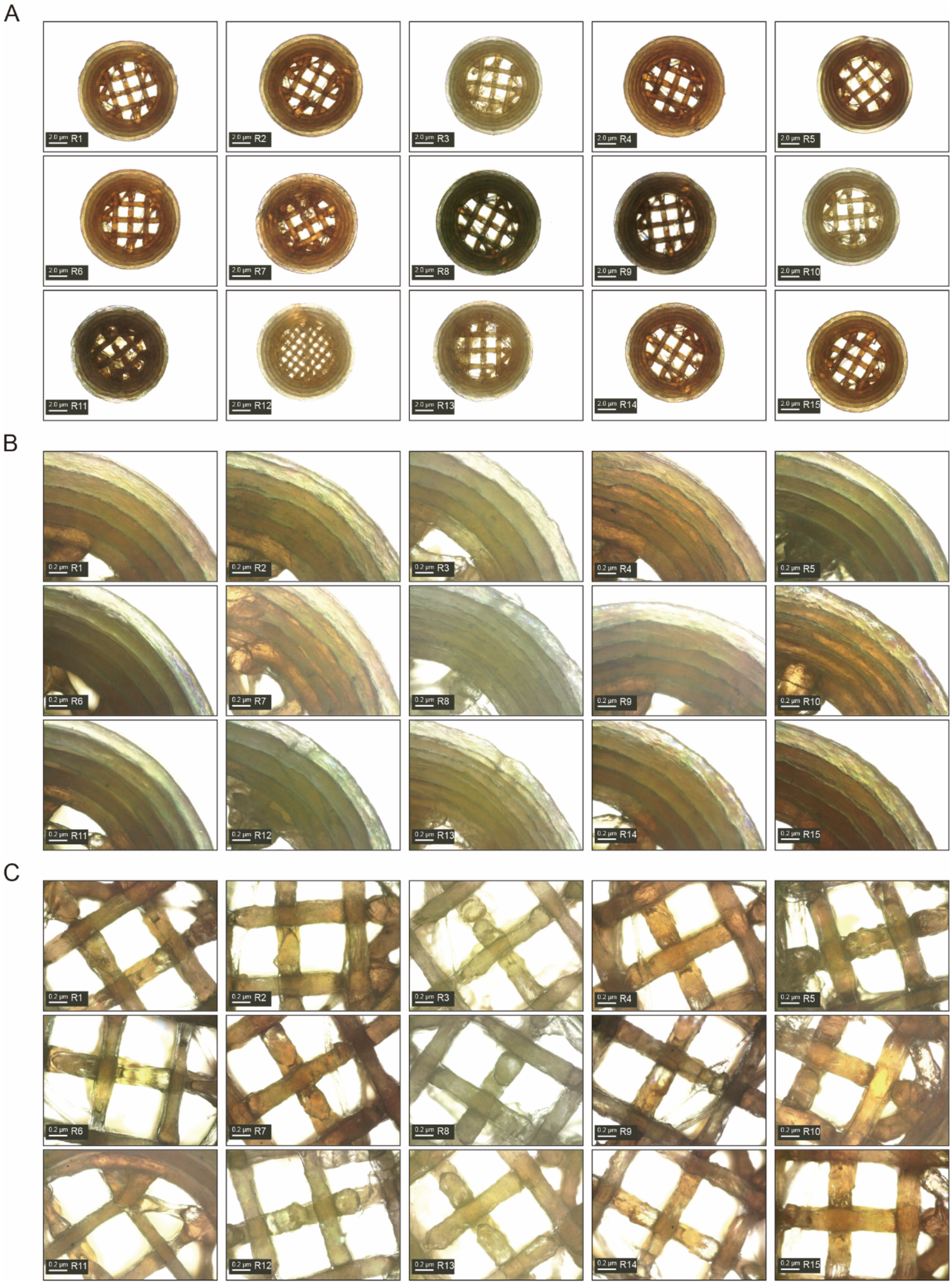

Figure S4. Micrographs of 3D printed tablets produced from DoE filaments. (A) Top view. Scale bar: 2.0 µm; (B) Shell; (C) Infill. Scale bar: 0.2 µm.

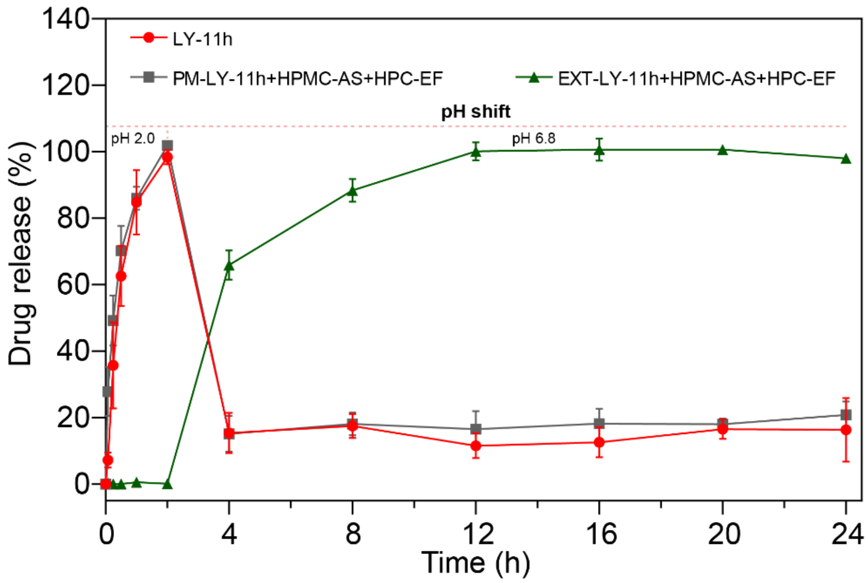

Figure S5. pH-shift dissolution profiles of pure LY-11h, the physical mixture, and the corresponding hot-melt extrudate.

Tables

Table S1. Box-Behnken design of 15 groups.

Group	Temperature (℃)	Screw speed (rpm)	Drug loading (%)	Distance (mm)	Force (gf)	Amorphization (%)	Tg (℃)	Torque (N·m)
1	160.00	100.00	17.50	1.9	2624.81	96.87	70.5	2.3
2	160.00	200.00	17.50	1.97	1965.34	94.96	68.9	2.8
3	160.00	150.00	5.00	2.28	3152.08	100	77.5	3.2
4	140.00	150.00	17.50	2.3	2718.85	96.46	65.2	3.9
5	160.00	150.00	30.00	2.17	1921.58	94.88	63.7	2.1
6	120.00	150.00	30.00	1.26	1149.39	76.28	59.7	5.1
7	120.00	200.00	17.50	1.64	1500	85.26	62.5	5.2
8	140.00	200.00	30.00	2.06	1970	97.65	57.2	3.6
9	120.00	100.00	17.50	1.24	1921.92	78.96	64	4.6
10	120.00	150.00	5.00	1.93	2922.28	88.8	66.2	4.8

11	140.00	100.00	30.00	1.49	1949.35	89.9	64.1	3
12	140.00	200.00	5.00	2.1	2443.14	102	72.7	3.7
13	140.00	100.00	5.00	1.94	4183.74	100	69.5	3
16	140.00	150.00	17.50	2.24	2802.72	96.47	67.2	3.9
17	140.00	150.00	17.50	2.53	2514.47	96.34	65.9	4

Table S2. The fitting models of the correlation between individual factors (temperature, screw speed, and drug loading) and response.

response	Model	R^2	*p-value*	response	Model	R^2	*p-value*
Distance	Original	0.7259	0.0044	Force	Original	0.8524	0.0045
	Square Root	0.7703	0.0031		Square Root	0.8446	0.0042
	Natural log	0.8019	0.0023		Natural log	0.7045	0.0107
	Inverse Sqrt	0.8186	0.0018		Inverse Sqrt	0.4549	0.0295
	Inverse	0.8192	0.0016		Inverse	0.0638	0.1330
Amorphization	Original	0.8936	0.0002	Tg	Original	0.9557	0.0003
	Square Root	0.8954	0.0002		Square Root	0.9553	0.0003
	Natural log	0.8916	0.0002		Natural log	0.9518	0.0004
	Inverse Sqrt	0.8823	0.0003		Inverse Sqrt	0.9450	0.0006
	Inverse	0.8675	0.0004		Inverse	0.9352	0.0009
Torque	Original	0.9155	0.0046				
	Square Root	0.8554	0.0177				
	Natural	0.7782	0.0447				

	log		
	Inverse Sqrt	0.6847	0.0810
	Inverse	0.5760	0.1155

Table S3. The mathematical models of correlation between the individual factors and distance.

	T	S	D	T*S	T*D	S*D	T^2	S^2	D^2
p-value	0.0002	0.0024	0.0019	0.0229	0.0058	0.0445	0.0009	0.0019	0.1294
Significance	***	***	***	*	**	*	***	***	NS
Code Equation	Distance = (7.15515-0.078185*T-0.016584*S+0.043066*D+4.44985E-005*T*S-2.53283E-004*T*D-5.85722E-005*S*D+2.53487E-004* T^2+3.42600E-005* S^2+1.65879E-004* D^2)								

Significance: $^{NS}p > 0.05$, $*p < 0.05$, $**p < 0.01$, $***p < 0.005$. T: temperature; S: screw speed; D: drug loading

Table S4. The mathematical models of correlation between the individual factors and force.

	T	S	D	T*S	T*D	S*D	T^2	S^2	D^2
p-value	0.0035	0.0011	<0.0001	0.4583	0.1261	0.0019	0.0011	0.0881	01779
Significance	*	***	***	NS	NS	***	***	NS	NS
Code Equation	Force=-21601.66235+372.04080*T+8.50840*S-265.73236*D-0.059387*T*S+0.54239 * T*D+0.70450*S*D-1.28236*T^2-0.065087*S^2+0.77182*D^2								

Significance: $^{NS}p > 0.05$, $*p < 0.05$, $***p < 0.005$. T: temperature; S: screw speed; D: drug loading

Table S5. Equilibrium solubility of pure LY-11h and extruded formulations prepared under different single-factor conditions. Values are expressed as mean ± SD (n = 3).

Investigated factor	Formulation/condition	pH 1.2 solubility (μg/mL)	pH 6.8 solubility (μg/mL)
Reference	Pure LY-11h	651.2 ± 25.3	2.8 ± 1.1
Drug loading	5% LY-11h	9.0 ± 0.1	224.1 ± 7.1
Drug loading	10% LY-11h	16.8 ± 2.8	239.1 ± 7.2
Drug loading	30% LY-11h	37.5 ± 3.3	228.4 ± 12.7
Drug loading	50% LY-11h	662.4 ± 40.8	226.1 ± 13.8

HPC-EF content	2.5% HPC-EF	7.0 ± 0.3	223.1 ± 8.3
HPC-EF content	5% HPC-EF	8.9 ± 0.3	232.3 ± 11.6
HPC-EF content	10% HPC-EF	7.7 ± 0.8	226.8 ± 5.6
HPC-EF content	30% HPC-EF	8.3 ± 0.5	202.4 ± 10.9
HPC-EF content	50% HPC-EF	8.1 ± 0.8	183.5 ± 9.4
HPMC-AS grade	HPMC-AS LG	24.4 ± 2.3	164.1 ± 11.9
HPMC-AS grade	HPMC-AS MG	12.9 ± 0.9	225.8 ± 9.6
HPMC-AS grade	HPMC-AS HG	9.0 ± 0.2	252.7 ± 12.4
HPMC-AS grade	HPMC-AS HF	11.4 ± 2.6	235.8 ± 7.7
Extrusion temperature	120 °C	303.9 ± 16.6	205.9 ± 14.4
Extrusion temperature	140 °C	10.9 ± 1.2	238.6 ± 13.3
Extrusion temperature	160 °C	10.1 ± 1.0	244.4 ± 0.9
Extrusion temperature	180 °C	2.39 ± 0.9	250.4 ± 1.3
Number of mixing sections	0 mixing section	7.9 ± 0.4	235.9 ± 10.1
Number of mixing sections	1 mixing section	6.7 ± 0.8	237.7 ± 13.0
Number of mixing sections	2 mixing sections	8.0 ± 0.1	238.2 ± 18.1
Screw speed	50 rpm	9.8 ± 0.8	241.2 ± 11.9
Screw speed	100 rpm	10.1 ± 0.1	244.5 ± 14.5
Screw speed	200 rpm	9.6 ± 0.4	243.8 ± 22.4
